# Conservation in the Iron Responsive Element Family

**DOI:** 10.3390/genes12091365

**Published:** 2021-08-30

**Authors:** Karl Volz

**Affiliations:** Department of Microbiology and Immunology, University of Illinois at Chicago, Chicago, IL 60612, USA; kvolz@uic.edu

**Keywords:** covariance model (CM), iron-responsive element (IRE), iron regulatory protein (IRP), messenger RNA (mRNA), pseudo-triloop (PTL), untranslated region (UTR)

## Abstract

Iron responsive elements (IREs) are mRNA stem-loop targets for translational control by the two iron regulatory proteins IRP1 and IRP2. They are found in the untranslated regions (UTRs) of genes that code for proteins involved in iron metabolism. There are ten “classic” IRE types that define the conserved secondary and tertiary structure elements necessary for proper IRP binding, and there are 83 published “IRE-like” sequences, most of which depart from the established IRE model. Here are structurally-guided discussions regarding the essential features of an IRE and what is important for IRE family membership.

## 1. Introduction

### 1.1. IRE Function

An iron responsive element (IRE) is a cis-acting messenger RNA (mRNA) regulatory motif with a unique, strongly conserved stem-loop sequence and structure. IREs are recognized by the iron regulatory proteins IRP1 and IRP2 in a very specific manner to modulate the fate of the IRE-containing mRNAs by either blocking their translation, or by stabilizing them [1,2].

### 1.2. IRE Family

A macromolecular family could be defined as a group of molecules evolutionarily related in sequence or structure that descended from a common ancestor and execute similar functions. Here I review the collection of RNA sequences that constitute the IRE family. The “family” nature of IRE structure and function was apparent in the first observations of the regulatory role of the IRE stem loop in the late 1980s [3,4]. This work is based on those and all following primary reports of “IRE” and “IRE-like” sequences, and especially builds on advances in the sequence analysis of IRE family organization [5,6,7,8,9,10,11].

## 2. Background

### 2.1. Two Applied Criteria

The goal here is to use the existing knowledge of IRE structure and function to reassess the 93 reported IRE sequences to date, and to contribute to an improved IRE ‘archetype’ for ascertaining family membership. The approach is to classify every published IRE-related RNA sequence as either “IRE” or “IRE-like”, using two main criteria: a motif-matching quality score from the bioinformatic program SIREs (Searching for IREs, [8]), and a log-odds score for a profile match by the program Infernal (Inference of RNA Alignments, [12]).

### 2.2. Classic IRE Model

**Figure 1:**
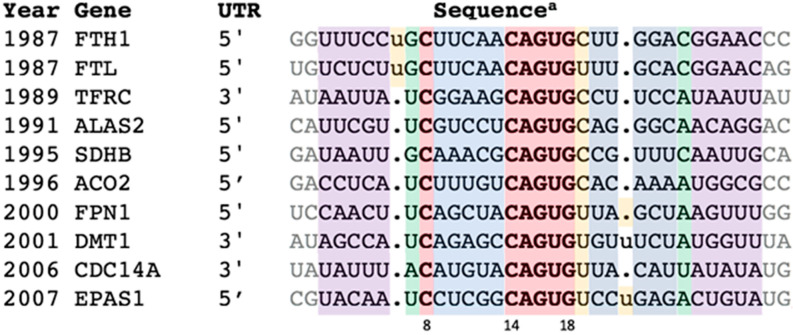
Representative IREs of genes regulated by the IRE:IRP system. ^a^ All sequences from *H. sapiens* except SDHB from *D. melanogaster*. Features color coded as in Figure 2. The extended set of orthologues is in Table S1, and alignment details in Figures S1 and S2. References: FTH1: [3,4,13,14,15]; FTL: [3,13,14,15]; TFRC: [16]; ALAS2: [5,17,18]; SDHB: [19,20]; ACO2: [21,22]; FPN1: [23,24,25,26]; DMT1: [27]; CDC14A: [28]; EPAS1: [29].

This reinvestigation began by reverting to the group of verified IREs—the ten IRE-containing genes (in order of discovery) FTH1, FTL, TFRC (b-d), ALAS2, SDHB, ACO2, FPN1, DMT1, CDC14A, and EPAS1—labeled here as “IREs” (Figure 1).

Building an IRE covariance model (CM) started with retrieving a few (two to six) closely-related orthologues for each of the ten confirmed IRE types from GenBank [30]. The resultant 38 entries were aligned without gaps except for the ferritin N6 and the DMT1/EPAS1 N21 bulges (Table S1, Figure S1). This seed group of just the “classics” contained only proven family members. High conservation (84% on average) within each type of IRE sequence (FTH1, FTL, TFRC, etc.) should ensure that they are orthologues, while greater inter-type variation (overall percent identity 46%, Figure S2) should produce a more accurate CM [31]. The characteristics of this group—the conservation, covariation, and secondary structure—were taken as the definition of the IRE.

### 2.3. IRE-Like” Sequences

The second group comprised all published RNA sequences claimed to be IRE-related but not represented in the “IRE” group. There were 83 such sequences, with publication dates ranging from 1993 to 2021. The current versions of their GenBank entries were retrieved and tabulated (Table S2) according to the conserved characteristics of the “IRE” group. Each sequence was scored with the programs SIREs [8] and Infernal [12], the latter using the CM built with the 38 sequences from the “IRE” group (Table S1). Any “IRE-like”sequence could be considered “classic” if it scored on both tests and had also demonstrated IRP-based regulatory activity in situ and/or in vivo, but none did. (Most “IRE-like” sequences in Table S2 were discovered during algorithm/program development, were not tested with biological assays, and were fairly presented without claims of functionality).

## 3. Discussion

### 3.1. Scoring Results

All 93 sequences (10 from Table S1 and 83 from Table S2) were scored by the programs SIREs [8] (http://ccbg.imppc.org/sires/ (2020–2021), accessed on 26 August 2021) and Infernal [12]. SIREs evaluates each sequence according to previously determined IRE:IRP binding- and RNA folding-properties and returns a nominal stringency level score of High, Medium, Low, or No Results. Infernal returns a numerical log-odds score (if above threshold) after aligning each sequence with a user-specified CM.

Interpretation of the two programs’ outputs was kept very simple: score or no score. No results from either program meant that the sequence being tested did not rank above the programs’ significance levels. As expected, 100% of the “IRE” group scored in both tests (Table S1, Columns G and H). The performance of the “IRE-like” sequences was far worse: although the majority (61%) returned a score from SIREs, most (80%) did not score above Infernal’s reporting threshold (lower settings had no effect). This is of concern because an Infernal test with this calibrated CM is an objective evaluation of a sequence’s potential to fold into the classic IRE stem-loop. It is also disconcerting that 30% of the “IRE-like” sequences were scoreless on both SIREs and Infernal (Table S2, Columns G and H). 

For the record, Figure 1 contains one sequence per “classic” IRE type, similar to the standard sequence alignment table often found in IRE review articles. Table S1 is an expansion of Figure 1 to two to six homologues per “classic” IRE type for building the CM. Table S2 has all the “IRE-Like” information; Column E is a useful visualization of the “IRE-like” sequences all together in a single accessible format. Shell scripts are provided for retrieval of the sequences from Genbank [30] in Supplementary Materials.

### 3.2. IRE Stem-Loops and the PTL Motif

**Figure 2:**
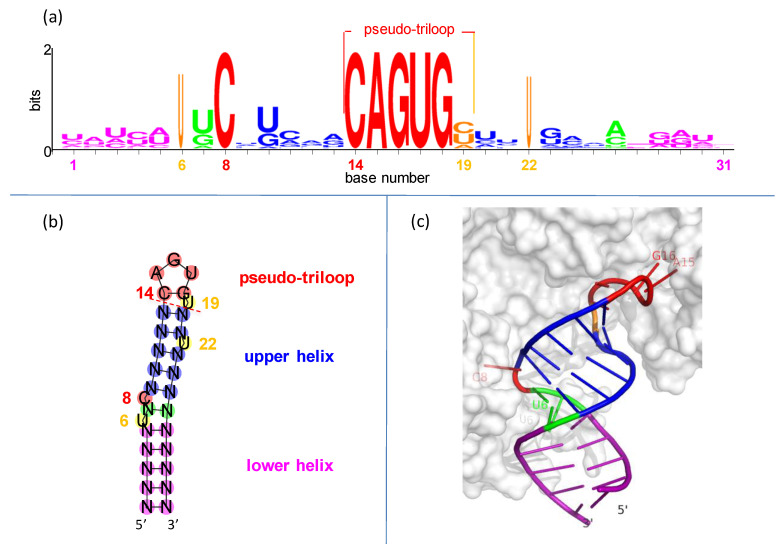
Conservation and structure in iron responsive elements (IREs). (**a**) IRE representation in seq-logo format [37]. Sequences are from Table S1. Symbol height shows the conservation at each position, while widths are proportional to the fraction present. (**b**) Secondary structure of hypothetical IRE with conventional number scheme as in (**a**). (**c**) Representative three-dimensional structure of IRE in complex with iron regulatory protein-1 (IRP1) [38]. The only bases making sequence-specific contacts with the IRP1 protein are C_8_ (**middle left**), and A_15_, G_16_, and U_17_ (**upper right**).

IREs are built on simple RNA hairpins. Hairpins, the most common RNA elements [32,33,34], are useful handles for binding by regulatory proteins [35,36]. Each IRE is only ~30 nucleotides long, folded into a hairpin of two short helices separated by a bulged C and topped with a conserved, six-nucleotide loop (Figure 2).

The IRE hexaloop is conventionally numbered 14 through 19. There is a special cross-loop (or transloop) base pair between bases 14 and 18 [39,40], making it plus the three intervening nucleotides 15–17 look like a triloop. This leaves the trailing 3′ base in position 19 hanging all by itself. The whole assembly of nucleotides 14–19 is called a pseudo-triloop motif, or PTL (it is also a type of lone-pair triloop, or LPTL [41,42]; “PTL” is the preferred term in the iron-regulation and virology fields [43,44]). “Pseudo” indicates that in some cases (not including IREs), a real triloop can be substituted for the PTL motif and retain function [43,44]. The PTL motif is 1–3 kcal-mol^−1^ more stable than expected [45,46].

### 3.3. One Type of IRE PTL Cross-Loop

In the IRE PTL, the cross-loop is made by nucleotides C14 and G18 and rests on base pair N13-N20 of the upper helix (Figure 2). It seems that the purpose of the PTL cross-loop is entirely structural, to impart conformational restraint on the presentation of the three recognition nucleotides to the binding proteins (see graphical demonstration in Figure S3). 

Statistically, the C-G base pair is highly favored in PTL cross-loops: C14 and G18 are two of the six invariant nucleotides in the IRE seed families of the Rfam database (RF00037 and RF02253 in https://rfam.xfam.org/ (2020–2021) accessed on 26 August 2021). This is likely due to the exceptional thermodynamic stability of a C-G closing pair in all stable RNA hairpins [34,47]. The C14-G18 of an IRE could be thought of as the closing base pair for the upper helix, although we conceptually group it with the PTL motif (Figure 2b; note that IRE PTLs do not seem to have a preference in the base pair preceding the cross-loop, N13-N20 ([48] and this work, Figure 2a), but viral PTLs strongly prefer yet another C-G pair there [44]).

This requirement for a C-G cross-loop in PTLs is underrepresented in IRE prediction software. This may have happened when early sequence selection-amplification and mutagenesis studies suggested broad tolerance for cross-loop base pairs, where U-A, G-C, and even G-G were found adequate for IRE-IRP binding [39,49,50,51]. These alternative pairs were considered atypical but acceptable, thus incorporated in IRE prediction routines (e.g., [8,52]). However, later analyses argued that IRE candidate screens based on in vitro binding alone are too permissive, and need corroboration with in situ and/or in vivo regulation evidence [53,54]. One naturally occurring IRE mutation vividly demonstrates the latter point: In human FTL IRE, a C→T mutation at position 14 created a U14-G18 cross-loop variant that could “compete effectively” with wild-type IRE in IRP gel-shift assays [55], but still caused a “severe phenotype” [56]. Put simply, an IRE with a U14-G18 cross-loop measurably binds IRP in vitro, but does not regulate in vivo. Thus

IRE Feature #1: An IRE should have a CG base pair at positions 14–18.

### 3.4. Three IRE PTL Recognition Bases

The IRE is a highly specific recognition element for the two IRP proteins. The key contacts for recognition in the apical loop are A_15_, G_16_, and U_17_. They must be conserved to retain the high affinity binding to IRP, which is absolutely necessary for regulatory function. The structural reasons for the conservation of A_15_, G_16_, and U_17_ are as follows.

A_15_ fully extends to make a hydrogen bond with S_371_ in all IRE:IRP1 complexes [38,57,58]. Using human ferritin IRE again as an example, there are no normally functioning IRE variants at position 15: variants A_15_→G and A_15_→U (in ferritin L and ferritin H, respectively) cause dysregulation and disease [59,60,61]. A spontaneous A_15_→C FTH1 mutation in humans has not yet been reported, but in vitro, that substitution fared worse than A_15_→U in competition assays with native FTH1 IRE [49]. By reasonably assuming that all IREs are recognized by IRP1 in the same way in the same binding site, an adenine should be retained in position 15.

Guanine has ideal H-bond-accepting properties [62,63] for G16 to make contact with K_379_ in the IRE binding cavity of IRP1 [38,57,58]. Cytosine does not have this bonding capability (nor does adenine), so it is understandable that the naturally occurring G_16_→C mutation in human ferritin L causes disease through loss of regulation [64]. The only functionally conserved substitution of guanine might be uracil, but G_16_ happens to be invariant in the published IRE seed alignments (RF00037 and RF002253 in Rfam https://rfam.xfam.org/ (2020–2021) accessed on 26 August 2021; see [9,11]).

U_17_ forms a hydrogen bond with the sidechain of R_269_ in all structures of IRE-IRP1 complexes [38,57,58]. This bond is critical for IRE-IRP regulation. Similar to the above discussion for G_16_, only a uracil could form that bond in that tight space [38,57,58], so U_17_ might be expected to be invariant among IREs. Early work considered adenine acceptable at position 17 only because of its presence in IRE A of the transferrin receptor (TFRC) 3′ UTR [65]. We now know that TFRC IRE A is not a site of IRP1 interaction [66], so it is reasonable to conclude that position 17 in functional IREs is only uracil.

To summarize, A_15_, G_16_, and U_17_ on the apex of the IRE PTL motif make the majority of the IRE hydrogen bonds with IRP1. Of course hydrogen bonds do not account for binding strength, but they do drive specificity [67]. These interactions are necessary for proper IRP-IRE regulation. Thus:

IRE Feature #*2:* An IRE should have an AGU triplet at positions 15-16-17.

If this apical loop is anything other than A_15_G_16_U_17_, weaker IRP-IRE binding may occur, but it may not support in vivo regulatory function.

### 3.5. A Critical IRE PTL Scaffold Position

The last nucleotide in the IRE PTL motif is N_19_, all by itself, pinched-out between the cross-loop and upper helix (Figure 2). It has no identified function. In nine appearances in multiple crystal structures [38,57,58], the N_19_ base is solvent accessible, has no protein contacts, and occupies many conformations with relatively high atomic displacement parameters (see Figure S4). This conformational variability of the exposed N_19_ in the crystal structures agrees well with its high mobility observed by NMR [45,68]. These facts argue that the lone N_19_ base of an IRE is not important for IRP recognition: the N_19_ sugar-phosphate is a place holder, present only for backbone support of the IRE PTL motif (see [44]). When the entire N_19_ nucleotide is removed, the remaining C_14_AGUG_18_ folds as a simple triloop, with complete loss of function [48,49]. (When the binding contribution of the last exposed base of a PTL motif is explored by deletion, outcomes vary depending upon how the RNA interacts with its binding protein. For IRE PTLs, deletion of N_19_ lowers the IRE-IRP binding affinity by 10^2^–10^3^ and destroys regulatory function [48,49]. This means that a ∆N_19_ IRE cannot assume the tight IRP-binding conformation. Results are the opposite for some viral PTLs, where equivalent deletions have no functional effects [69,70,71,72]. This interchangeability of PTLs with triloops in the viral systems means they present the same way to their cognate protein(s).

Therefore, N_19_ has to be present in IREs, with the only requirement that it not be complementary to N_14_. Using the typical C_14_AGUG_18_N_19_ IRE PTL as example (see cross-loop section above, and Figure 2b), if N_19_ were G it would out-complete G_18_ to pair with C_14_ and stack on to the stem helix, creating the tetraloop C_14_AGUGG_19_ that would not be recognized by IRP1/IRP2. Finally, it is preferable that the exposed base of N_19_ be pyrimidine and not purine, perhaps because of the lower solvation cost. Thus

IRE Feature #3: An IRE should have a U or C bulge at position 19.

For the 38 “classic” IRE sequences in Table S1, the frequencies of A/U/C/G at position 19 happen to be 0.16/0.39/0.45/0.00.

### 3.6. A Specific IRE Stem Recognition Base

A critical contribution to IRE-IRP regulation comes from the single cytosine bulge below the five base-pair IRE upper helix at position 8. In all structures of IRE-IRP1 complexes [38,57,58], C_8_ has the same extensive complementary interactions with protein backbone and side chains in a deep pocket of IRP1 domain 4 (see Figure 4B of [38]). The tight pocket seems custom-designed for a cytosine, and C_8_ is widely conserved. (Inexplicably, brachiopods, annelids, and arthropods have a guanine instead of a cytosine at IRE position 8 [7,73,74,75]. Their apparent normal IRE-IRP function with G_8_ IREs remains structurally unexplained. Presumably the IRPs of those organisms have compensating evolutionary adjustments (discussed in [58]). The position taken here is that cytosine is required at IRE position 8 unless the organism is closely related to the above exceptions.)

An early SELEX study argued that uracil or guanine can be substituted for the bulged C_8_, provided that other substitutions within the loop are also present [76]. Results from other studies disagreed: clean C_8_→U and C_8_→G substitutions show significant reductions in affinity to IRP1, and loss of function in vivo [49,77,78]. In addition, the SELEX study’s claim that “the C-bulge functions to orient the hairpin rather than directly contact the protein” is not consistent with the extensive protein contact in the three-dimensional structures of the IRE-IRP1 complexes [38,57,58].

Independent binding analyses came to the same conclusion that C_8_ is essential for productive IRE-IRP interaction [39,48,50]. Furthermore, the naturally occurring mutations C→A and C→T at position 8 of ferritin L cause disease [61,79,80,81], even though it was earlier reported that “C→A does not diminish IRE-BP binding significantly” [82] (like the U_14_-G_18_ cross-loop variant discussed above, this is another IRE mutation that measurably binds IRP in vitro, but does not regulate in vivo. This seems common in IRE research, where point mutations that only mildly impair IRP binding still cause disease [83]). Therefore, for the purposes of screening for IRE family membership, there is

IRE Feature #4: An IRE should have a bulged C at position 8.

### 3.7. Precise Three-Dimensional Spacing

The bases of C_8_ and the apical triad A_15_G_16_U_17_ are responsible for most of the ~two dozen nucleotide-specific contacts between IRE and IRP1 [38,57,58]. Together the C_8_ and the apical loop create a rigid “two-point” fashion of contact, separated by the critical distance and rotation provided by the intervening, five base-pair, A-form upper helix [38,48,82,84].

Thermodynamic contributions of multi-point interactions are notoriously greater than additive, and can result in extremely tight complex formation [85,86]. The K_d_ for FTH1 IRE-IRP1 is ~30 pM (Table S3). Although some of these critical four bases can be substituted while retaining intermediate-strength IRP binding, all four are necessary for true IRE-IRP regulation [49]. The important conclusion is that C_8_ and A_15_, G_16_, and U_17_ should all be present—with their precise relative spacing and twist—for an RNA sequence to bind to an IRP [48,49,78]. (DMT1 (SLC11A2) and EPAS1 IREs each have a characteristic extrahelical base in the 3′ side of their upper helix (Figure 2b and Figure S1, Table S1, [29,87]). Single bulges like these would not necessarily change the relative positions of the A_15_G_16_U_17_ PTL recognition triplet and C_8_. The original papers demonstrated that these extrahelical bases could be assigned positions following N_21_ or N_22_, locations not likely to interact with protein in an IRE-IRP1 complex (discussed in [88]). For simplicity, this work follows the practice of SIREs [8] (http://ccbg.imppc.org/sires/ (2020–2021) accessed on 26 August 2021) to group the DMT1 and EPAS1 bulges following N_22_.) Thus

IRE Feature #5: An IRE should have a five base-pair upper helix.

These five IRE Features are just explanations of the nature of IRE:IRP binding—they were not used in any scoring (the red highlighting of the differing nucleotides in Table S2 Column E are for illustrative purposes only). There are valid exceptions to these IRE Features, but they do not change the scoring results from SIREs and Infernal.

### 3.8. Additional Criteria for IRE Identification

Other characteristics of the IRE family—lower helix length and composition, bulges and insertions, mismatch locations, GU base-pair counts, folding free energies, etc.—are thought to contribute to their specificity in interacting with the IRP proteins [48,89], and are used quantitatively for testing new sequences. As emphasized above, the best systematic treatment of these and other IRE-specific properties in RNA sequence screening is the SIREs web server 2.0 ([8], http://ccbg.imppc.org/sires/ (2020–2021) accessed on 26 August 2021).

Minor characteristics potentially useful in considering prospective IRE sequences are as follows:

• IREs are restricted to metazoans [7], so all IRE-like sequences from prokaryotes and protozoans can be confidently excluded. This basic fact applies to 17% of the published ”IRE-Like” sequences (Table S2 Column C, red).

• IRE-IRP systems have broad phylogenetic distribution [7]. In general, functional RNAs show structural conservation among related species [90], so candidate IRE sequences should be present in closely related species, and critical sites should be conserved.

• IRE position in the mRNA can be diagnostic: IREs are predictably located in the 5′ or 3′ UTRs, and rarely in the coding regions (CDS) (the famous IRE exception being ACO2 [21]). Furthermore, there is no mechanistic explanation why cis-acting regulatory elements would be located in the negative strand. Thirty-five percent of the published “IRE-like” sequences have such questionable locations, and are highlighted in red in Table S2, Column F.

• IRE location within the UTRs of the mRNAs is critical; for 5′ UTRs, the distance of the IRE from the mRNA cap and AUG start site can influence regulatory effectiveness [28,29,91,92,93,94,95]. These IRE-to-cap and IRE-to-start distances may vary according to gene type (see the FTH1 IRE example in Figure S5), so could be used as weighting criteria in IRE family screening.

## 4. Conclusions

### IRE Family Reassessment

The 34 years of research in iron regulation have provided a detailed understanding of IRE structure and function. While in situ and in vivo tests provide the definitive evidence for IRE regulatory activity, much can be inferred from the gene and mRNA sequences, given the available software tools and their proper use. Such predictive analyses should be performed with care and sound judgment. This work shows that many IRE claims are unlikely to be true. The existing criteria for IRE family membership should be reexamined with this in mind. The goal here is to recommend a conservative reassessment of IRE-IRP structure and function.

## Data Availability

Not applicable.

## Supplementary Materials

The following are available online at https://www.mdpi.com/article/10.3390/genes12091365/s1, Figure S1: Multiple alignment of the 38 IRE sequences representing the “classic” IREs, Figure S2: Percent identity array for the 38 IRE sequences representing the “classic” IREs, Figure S3: Structural similarities and differences in PTLs from three unrelated sources, Figure S4: Positions of IRE N19 in 12 occurrences in five x-ray structures of IRE-IRP1 complexes, Figure S5: An IRE-centric view of FTH1 5′ UTRs, Table S1: 38 Representative Sequences For The Ten IRE-Containing Genes, Table S2: 83 “IRE-Like” Sequences, Table S3: Published Ftn IRE-IRP1 Binding Strengths, Table S4: Published PTL Structures With Coordinates Deposited In PDB, Script S1: Unix Script for Retrieval of IRE Sequences in Table S1, Script S2: Unix Script for Retrieval of IRE-Like Sequences in Table S2.
