# Supplementary material for: Conservation in the Iron Responsive Element Family
Source: Genes (Basel). 2021 Aug 30;12(9):1365. doi: 10.3390/genes12091365 (PMC8466369; doi:10.3390/genes12091365)
Supplement: Supplementary file 1 [file genes-12-01365-s001.zip › genes-1344373-supplementary.pdf]

Review

Conservation in the Iron Responsive Element Family

Karl Volz

Department of Microbiology and Immunology

University of Illinois at Chicago

Chicago, Illinois 60612

kvolz@uic.edu

All Supplemental Material (Tables S1-S4, Figures F1-F5, References, and Scripts S1-S2)

Table S1: Representative Sequences For The Ten IRE-Containing Genes

FTH1, FTL, TFRC, ALAS2, SDHB, ACO2, FPN1, DMT1, CDC14A, and EPAS1.

A	B	C	D	E	F	G	H	I
**Gene Access.Ver**	**Range**	**Organism**	**Gene Symbol**	**Sequence**	**Location**	**SIREs Quality**	**Infernal Score**	**Ref**
NM_002032.3	29-63	*Homo sapiens*	FTH1	GGGUUUCCuG**C**UUCAA**CAGUG**CUU.GGACGGAACCC	5'	High	31.2	1-5
NM_131585.1	3-37	*Danio rerio*	FTH1	AGGUUACCuG**C**UUCAA**CAGUG**CUU.GAACGGCAACC	5'	High	29.1	
NM_205086.1	29-63	*Gallus gallus*	FTH1	CGGGUUCCuG**C**GUCAA**CAGUG**CUU.GGACGGAACCG	5'	High	28.0	
NM_010239.2	83-117	*Mus musculus*	FTH1	CGGUUUCCuG**C**UUCAA**CAGUG**CUU.GAACGGAACCC	5'	High	33.8	
NM_001172847.1	2-36	*Cavia porcellus*	FTH1	CGGUCUCCuG**C**UUCAA**CAGUG**CUU.GGACGGAGCCC	5'	High	32.4	
NM_000146.4	23-57	*Homo sapiens*	FTL	CUGUCUCUuG**C**UUCAA**CAGUG**UUU.GCACGGAACAG	5'	High	22.9	1, 3-5
NM_010240.2	76-110	*Mus musculus*	FTL	CUGUGUCUuG**C**UUCAA**CAGUG**UUU.GAACGGAACAG	5'	High	30.4	
NM_022500.4	28-62	*Rattus norvegicus*	FTL	CUGUAUCUuG**C**UUCAA**CAGUG**UUU.GGACGGAACAG	5'	High	29.7	
NM_174792.4	79-113	*Bos taurus*	FTL	CUGUCUCUuG**C**UUCAA**CAGUG**CUU.GGACGGAACAG	5'	High	30.7	
NM_001128148.3	3286-3319	*Homo sapiens*	TFRC b	UAUAAUUA.U**C**GGAAG**CAGUG**CCU.UCCAUAAUUAU	5'	High	32.1	6
NM_001128148.3	3690-3723	*Homo sapiens*	TFRC c	ACACAUUA.U**C**GGGAG**CAGUG**UCU.UCCAUAAUGUA	5'	High	30.4	
NM_001128148.3	3755-3788	*Homo sapiens*	TFRC d	UGUCUGUA.U**C**GGAGA**CAGUG**AUC.UCCAUAUGUUA	5'	High	30.7	
NM_011638.4	3336-3369	*Mus musculus*	TFRC b	GAUAAUUA.U**C**GGAAG**CAGUG**CCU.UCCAUAAUUAU	5'	High	30.7	
NM_011638.4	3715-3748	*Mus musculus*	TFRC c	UCACAUUA.U**C**GGGAG**CAGUG**UCU.UCCAUAAUGCA	5'	High	31.6	
NM_011638.4	3780-3813	*Mus musculus*	TFRC d	UGUCUAUA.U**C**GGAGA**CAGUG**AUC.UCCAUAUGUUA	5'	High	32.5	
NM_000032.5	6-39	*Homo sapiens*	ALAS2	UCAUUCGU.U**C**GUCCU**CAGUG**CAG.GGCAACAGGAC	5'	High	27.2	7-9
XM_002125731.4	53-86	*Ciona intestinalis*	ALAS2	CUACUAGU.U**C**GUCCU**CAGUG**AAG.GGCAAUUGUCA	5'	High	29.4	
NM_131682.2	1-31	*Danio rerio*	ALAS2	GAAGU.U**C**GUCCU**CAGUG**CAG.GUCAACAGCTG	5'	High	17.9	
NM_001035103.2	7-40	*Bos taurus*	ALAS2	UCGUUCGU.U**C**GUCCU**CAGUG**CAG.GGCAACAGAAC	5'	High	28.6	
NM_057753.5	78-111	*Drosophila melanogaster*	SDHB	CGAUAAUU.G**C**AAACG**CAGUG**CCG.UUUCAAUUGCA	5'	High	32.1	10-11
XM_002004955.3	96-129	*Drosophila mojavensis*	SDHB	AACAAAUU.G**C**AAACG**CAGUG**ACG.UUUCAAUUUCA	5'	High	26.6	
NM_001098.3	5-38	*Homo sapiens*	ACO2	CGACCUCA.U**C**UUUGU**CAGUG**CAC.AAAAUGGCGCC	5'-CDS	High	28.9	12-13
XM_844073.5	73-106	*Canis lupus familiaris*	ACO2	CGACUUCA.U**C**UUUGU**CAGUG**CAC.AAAAUGGCGCC	5'-CDS	High	29.0	
XM_009859766.3	145-178	*Ciona intestinalis*	ACO2	UUGGGUCA.U**C**UUUGU**CAGUG**AAC.AAAAUGGCAUC	5'-CDS	High	29.3	
NM_014585.6	95-128	*Homo sapiens*	FPN1 (SLC40A1)	UUCCAACU.U**C**AGCUA**CAGUG**UUA.GCUAAGUUUGG	5'	High	34.5	14-17
NM_131629.1	78-111	*Danio rerio*	FPN1 (SLC40A1)	CUCCGACU.U**C**AGCUA**CAGUG**AUA.GCUAAGUUUGG	5'	High	32.1	
NM_001012913.1	102-135	*Gallus gallus*	FPN1 (SLC40A1)	UUCCGACU.U**C**AGCUA**CAGUG**CUA.GCUAAGUCGGG	5'	High	32.1	
NM_001174125.2	1815-1849	*Homo sapiens*	DMT1 (SLC11A2)	GGUAGCCA.U**C**AGAGC**CAGUG**UGUuUCUAUGGUUUA	3'	Medium	28.2	18
XM_004400823.1	1843-1877	*Odobenus rosmarus divergens*	DMT1 (SLC11A2)	UAUAGCCA.U**C**AGAGC**CAGUG**UGUuUCUGUGGUUUA	3'	Low	24.8	
XM_013508458.1	1401-1435	*Chinchilla lanigera*	DMT1 (SLC11A2)	GGUAGCCG.U**C**AGAGC**CAGUG**CGUuUCUAUGGUUUA	3'	High	25.6	
NM_003672.4	2534-2567	*Homo sapiens*	CDC14A	UUAUAUUU.A**C**AUGUA**CAGUG**UUA.CAUUAUAUAUG	3'	High	31.1	19
XM_003585862.5	2189-2222	*Bos taurus*	CDC14A	UUACAUUU.A**C**AUGUA**CAGUG**UUA.CAUUAUGUAUG	3'	High	29.1	
NM_001134856.1	2627-2660	*Rattus norvegicus*	CDC14A	CUGCAUUU.A**C**AUGUA**CAGUG**UUA.CAUUAUAUAUG	3'	High	31.7	
NM_001430.5	70-104	*Homo sapiens*	EPAS1 (HIF2a)	CCGUACAA.U**C**CUCGG**CAGUG**UCCuGAGACUGUAUG	5'	High	31.8	20
NM_001005647.1	98-132	*Xenopus tropicalis*	EPAS1 (HIF2a)	CUGUACAA.U**C**CUCAG**CAGUG**CCCuGAGACUGUAUA	5'	High	31.0	
XM_011603052.1	146-180	*Takifugu rubripes*	EPAS1 (HIF2a)	CUGUACAA.U**C**CUCGG**CAGUG**UUCuGAGACUGUACG	5'	Medium	31.2	
XM_006007491.2	101-135	*Latimeria chalumnae*	EPAS1 (HIF2a)	CUGUACAA.U**C**CUCGG**CAGUG**UUCuGAGACUGUAUA	5'	Medium	31.5	
NM_001039806.2	112-146	*Danio rerio*	EPAS1 (HIF2a)	CUGUACAA.U**C**CUCAG**CAGUG**UUCuGAGACUGUACA	5'	Medium	28.8	
				* *****				

Table S2: "IRE-Like" Sequences

A	B	C	D	E	F	G	H	I
**Gene Access.Ver**	**Range**	**Organism**	**Gene Symbol**	**Sequence b**	**Location**	**SIREs Quality**	**Infernal Score**	**Ref**
NM_001063.4	1-33	*Homo sapiens*	TF (transferrin)	ACAGAAGCGAGUCCGACUGUGCUCGCUGCUCAG	5'	—	—	1
S57264.1	788-822	*Gallus gallus*	ALAS-E	CCAGUGCGUUCGUCCUCAGGGCGGGGCAACGGCAC	3'	Low	—	2
NM_013116.2	500-534	*Rattus norvegicus*	TSHb	AAUGCAAUCUGGUUAAAUGUGUUUACCUGGAAUAG	3'	—	—	3
M29730.1	244-278	*Escherichia coli*	*FepB* (ferrienterobactin)	UGCCCCCUCUCCCUUCCAGGGUGAGGGCUCGGGUG	CDS?	Low	—	4
M30785.2	1260-1294	*Escherichia coli*	*HemA* (ALAS)	AUUGAUCUUACGCAUCCUGUAUGAUGCAAGCAGAC	5'	—	—	4
J01611.1	2644-2678	*Escherichia coli*	*FrdB* (fumarate reductase)	CACCGCAUAGCGCAUUCUAUGAAGUGCCUUAUGAC	neg str	—	—	4
NC_000964.2	3913300-3913334	*Bacillus subtilis*	*qoxD*	AGCAAAAAACCCUCUUCAGUGGAAGAGGGUUUUUU	neg str; 3'	Medium;Low	—	4
NC_000964.3	182348-182382	*Bacillus subtilis*	*feuA*	ACAUACAAAACUAAUUCAGAGUAGGUUUUUGUGUC	CDS	—	—	5
NM_010403.2	1912-1946	*Mus musculus*	hao1	CAUUUAUAGUCACAUUCAGUGUAAAGUACAUAUUU	3'	Medium;Low	16.2	5
NM_005006.6	59-93	*Homo sapiens*	NDUFS1	CUCCAGGCCCGGCUGACAGAGUUAGCCGAGGCCGC	5'	—	—	6
NM_014117.3	58-92	*Homo sapiens*	PRO0149 (C16ORF72)	UCAGCGGCGGCGGCGGCAGUGGGGAGGCCGCAGCG	5'	—	—	7
AF059611.1	94-128	*Homo sapiens*	NRPb/ENC-1	CCUUCCGCGGCGCCUGCAGUGGCAGGCUUGCUCUG	5'	—	11.1	7
NM_001079802.2	4538-4572	*Homo sapiens*	fukutin/FCMD	AACACAGUAUCUUUAACAGUGCUUGAGAUGCUUAA	3'	Medium;Low	18.3	7, 8
NM_000484.4	70-104	*Homo sapiens*	APP	GCGGUGGCGGCGCGGGCAGAGCAAGGACGCGGCGG	5'	—	—	9
LR131493.1	505294-505328	*Plasmodium falciparum*	PfIRE1	AUAUGUCCAACUUAUAAAGUUAUAUAAUUAUUAAU	3'	Low	—	10
LR131481.1	390177-390211	*Plasmodium falciparum*	PfIRE2	ACAACGUGUUCUUGUUAAGUUGAACAAAAAAUAUG	5'	High	—	10
LR131482.1	187714-187748	*Plasmodium falciparum*	PfIRE3	UUUAUUUAUUCUUAAUGAGUCUAUUAAAAAAACAA	neg str	—	—	10
X90566.1	6-40	*Pacifastacus leniusculus*	Ferritin	AGACGCUCCGGGUCGCCAGUGUGUGAACGAGCUCC	5'	—	13.0	11
XM_027373946.1	214-248	*Penaeus vannamei*	Ferritin	GAGUGCUCCGGGUCACCAGUGUGUGGACGAGUACC	5'	—	14.2	12
DQ207752.1	17-51	*Periserrula leucophryna*	Ferritin	CUGUAUCUUGGGACGUCAGUGUGCGUACGGAUCGG	5'	—	12.3	13
NM_014826.5	7009-7043	*Homo sapiens*	CDC42BPA (MRCKa)	CUGUAGAAAACACUUGCAGAGCCAGGUUUUGCUGG	3'	Medium;Medium	—	14
NM_079217.3	213-247	*Drosophila melanogaster*	*S6K*	CGUUGUGCGUGCGUCGCAGUGUUGGUGCGUGUGCC	5'	—	—	15
NG_029489.1	108892-108926	*Homo sapiens*	*BRF1*	GGGGCAGGGGCCGGUGCAGAGCCACUGUCUGUGUU	3'	Medium	—	15
AK091913.1	1929-1963	*Homo sapiens*	*FLJ34594*	AAGGAGCUCCCUGACCCAGAGAGGUUAAGGGUUUG	3'	Medium; Low	—	15
AK126633.1	3183-3217	*Homo sapiens*	*FLJ44675*	UGUGAAGAUUCUUUGGCAGUGUCCAAGAAUUAUCU	3'	Medium;Low	20.0	15
NM_000345.4	182-216	*Homo sapiens*	SNCA	AGUGGCCAUUCGACGACAGUGUGGUGUAAAGGAAU	5'	—	—	16-18
NC_000962.3	4403226-4403260	*Mycobacterium tuberculosis*	*trxC*	UGCGCUGUGGCGACCGCAGUGCGGCCGUCACCGAG	3'	—	—	19
NC_000962.3	3023462-3023496	*Mycobacterium tuberculosis*	*ideR*	GGUAUGCCCGCCGCGCCAGCGGCGGGCAUACCGCU	5'	—	—	19
AY679763.1	2722-2756	*Trichomonas vaginalis*	*cp4*	UACACAUGUUCGUUCAGGCACAUGAGCAGAAAGCC	neg; CDS	—	—	20
AY371180.1	761-795	*Trichomonas vaginalis*	*cp12*	UUUAAACGUAUUUAAUUGAUUGCGAAAUUUCUUUU	3'	—	—	21
NM_016633.4	454-488	*Homo sapiens*	AHSP	ACACCUCAAUAAAGACCAGUGCUGGUUUUGUUGGA	3'	—	—	22
XM_017024763.1	1038-1072	*Homo sapiens*	PHB	GCUCCUCCAGCUGCCCCAGUGAGGGCCCACCCUGC	CDS; 3'	High	11.9	23
NM_005329.2	3307-3341	*Homo sapiens*	*HAS3*	CUGUUCUCAGCGUUUUGAGUUUAAAACCUGGGAUC	3'	—	—	8
NM_001313893.1	2543-2577	*Homo sapiens*	*HMGB1*	UUGUUGACAUUAGUCUCAGUGAAGACUAUGAAAAU	3'	—	16.0	8
NM_198560.3	4510-4544	*Homo sapiens*	*LHFPL4*	ACCUUUCUCCAGCUUCCAGUGUGAACUGAGAAAGA	3'	—	—	8
NM_012214.3	2513-2547	*Homo sapiens*	*MGAT4A*	UGCCAUUUUUCAAAUGCACUGCCAUUUAAGAUUGA	3'	—	—	8
NM_001197026.2	6823-6857	*Homo sapiens*	*PLEKHA8*	UUAAUCACUUCUGUUCCAGAGUGAACAAAUGUUUU	3'	Medium	—	8
NM_000551.4	806-840	*Homo sapiens*	*VHL*	CAAAGUGUCUCAUUCUCAGAGUAAAAUAGGCACCA	3'	High	—	8
NM_006870.4	238-272	*Homo sapiens*	*DSTN*	CUGUCAUUUUUUGUCUCAGUGCAGACAAAAAGUGC	CDS	—	19.9	8
NM_001977.4	3082-3116	*Homo sapiens*	*ENPEP*	AACAUAGAAACACCAUCAGAGAAUGGUUUUUUAAU	CDS	High	—	8
NM_001080462.3	600-634	*Homo sapiens*	*TMEM202*	AUUAUCUUAACUGGUGCAGUGACAUCUUUUACAUG	CDS	High	17.9	8
NM_001082968.2	359-393	*Homo sapiens*	*TOM1L2*	ACCGAGAUUUCAUCGACAGUGUUCUGGUCAAAAUU	CDS	—	12.7	8
NM_001025573.2	954-988	*Mus musculus*	Slc23a4	UUCCGCUUCCCUUACCCAGGGCAGUGGGGCCUCCC	CDS	Low	—	24
NM_178629.6	2143-2177	*Mus musculus*	Crppa	UGGGCAAACUCUUGCCUAGUAUGGAUAGAGCGACA	3'	High	—	24
NM_173737.2	133-167	*Mus musculus*	Hmces	CCGGCGGCGGCUCCCGCAGUGGAGGGACCCCGACA	CDS	Medium	—	24
NM_001081436.2	1060-1094	*Mus musculus*	Ino80d	UUGUAUAAGCCGCCUGCAGAGACUGGUGAAACUGU	CDS	Medium	—	24
AK134743.1	818-852	*Mus musculus*	cDNA; clone:6330544L24	GUGAGAGUAGCUUUUUGAGUGUGUAAGCCUACAUU	N/A	Low	—	24
NM_173505.4	1945-1979	*Homo sapiens*	ANKRD29	UCUUGAGUUUCCAACACCGUGCUGCUUGAUAGAAU	3'	Medium	—	24
NM_183170.2	162-196	*Mus musculus*	Mpv17l2	GCGCGCGCGGCCUGGACAGAGAUUCAGCGCGCGGC	CDS	—	—	24
NM_023158.7	1342-1376	*Mus musculus*	Cxcl16	GCAGGCUCGUCUCCAUCAGUGAAUGGAACCUGAGC	3'	High	13.2	24
NM_011807.3	2320-2354	*Mus musculus*	Dlg2	ACUACGAAGUCGACGGCAGAGACUAUCACUUUGUC	CDS	High	—	24
NM_001364.3	4669-4703	*Homo sapiens*	DLG2	AGAGAUAGAGCCAUCAGAGUGAUGGGGCUUCUUCA	3'	Medium	—	24
NM_010118.3	1238-1272	*Mus musculus*	Egr2	GGCAGUGGGUCUGCAGCAGUGACUGCCACCCCUUA	CDS	High	13.6	24
NR_028406.2	469-503	*Homo sapiens*	FXYD5	CUCCUUGCGUCAGUCCCAGUGAGGGAUAAGCGCCU	5'	Medium	—	24
NM_019994.5	5172-5206	*Mus musculus*	Ralgapa1	UUACACUCUCCAGAUUCAGAGCGGUCUUCUAAACU	CDS	High	—	24
NM_001003719.2	710-744	*Mus musculus*	Ralgapa1	UCGUCGGGACCCGGCCGGGAGGGGCCGCGGCGGCC	5'	Medium	—	24
NM_008184.3	1084-1118	*Mus musculus*	Gstm6	AUGCAGGCUCCAUCUCCAGAGUGGGAAGGCCCAGU	3'	High	—	24
NM_133994.3	955-989	*Mus musculus*	Gstt3	GACUUGGUGGCCAUCACAGAGCUGAUGCAUCCUGU	CDS	High	—	24
NM_013755.4	473-507	*Mus musculus*	Gyg	UCAGCAGCACCAGACCCAGGGUGGCCUGACUGUUU	CDS	Low	—	24
NM_004130.4	462-496	*Homo sapiens*	GYG1	UCAGCAGCACCAGACCCAGGGUGGCCUGACUGCUU	CDS	Medium	—	24
NM_004130.4	1275-1309	*Homo sapiens*	GYG1	AGAUGAGAGGCUUUUUUAGGAUAAGAGGUGAGAAC	3'	Low	—	24
NM_201531.4	3177-3211	*Mus musculus*	Kcnf1	AAAGGCUAAGCUGGGCCAGUGGGCCUAACCCUUUA	3'	High;Medium	—	24
NM_201531.4	4720-4754	*Mus musculus*	Kcnf1	UUUUGUGAAUCGUGAAUAGUACUUUACAUUCAAAA	3'	—	—	24
NM_013587.3	2272-2306	*Mus musculus*	Lrpap1	UUCCCAGAACCCUCAGCAGUGUCUGAGGCUCAGAG	3'	High	19.2	24
NM_173199.3	387-421	*Homo sapiens*	NR4A3	CGCUCACCGCCUCCGGGAGCCCCUGGGCUUGUACA	5'	Low	—	24
NM_016467.5	894-928	*Homo sapiens*	ORMDL1	AAGUUAAAGGGCAUCACAGUGAGGGUGUAGUAGAU	3'	Medium	—	24
NM_001101479.1	1151-1185	*Mus musculus*	Pabpc4l	UCUUUGGGUCCAUUUGCAGGGUUAAAGUGAUGCAG	CDS	Medium	—	24
NM_144828.2	573-607	*Mus musculus*	Ppp1r1b	UUCCUGGGUGCGGGGACAGUGCUCCUCCUCCUCCU	5'	High	13.3	24
NM_024854.5	938-972	*Homo sapiens*	PYROXD1	UCAGUUACAGCUGAUACAGAGAUGUGGCCUGUCUA	CDS	Low	—	24
NM_021202.3	2043-2077	*Homo sapiens*	TP53INP2	AGGUCAGCGUGCAGAGCAGUGAUGCUGGAGGACAC	3'	Low	—	24
NM_019863.2	8-42	*Homo sapiens*	F8	CCUCGGCGGGCUGCCGCCGUGCCCGCGCCGGCUCC	5'; CDS	Medium; Medium	—	25
NM_020139.4	2276-2310	*Homo sapiens*	BDH2	AAUUCUGUAACAUUGUCAGGGCAAAGUUUUUUGUU	3'	Low	—	26
NM_002628.4	750-784	*Homo sapiens*	Pfn2	GGGGAGGGGGCAUGCCAAGUUGGGCAUCACUUUGU	3'	High	—	27
NM_007824.3	3086-3120	*Mus musculus*	Cyp7a1	GGGCUGGAGCAGGGUCCAGUGGAUGCUGUGCAGGU		—	—	28
NM_145005.6	104-138	*Homo sapiens*	C9ORF72_ALS	UGUGACAGUUGGAAUGCAGUGAUGUCGACUCUUUU	5'-CDS	—	—	29
NW_001915125.1	2650-2684	*Entamoeba histolytica*	Ehhmbp26	AAGUUGUAACUACAAUUUUAUAUGUCUCAAGUUAUACU[illegible]	5'	—	—	30
NW_001915125.1	3355-3382	*Entamoeba histolytica*	EHI_022250_3UTR	AAUAAAUUGAAUUAAUGUUCUCUUUAUU	3'	—	—	30
NM_012334.3	4649-4683	*Homo sapiens*	Myo10	GCCAGGAAUUCAUCGUGAGAGGAUGGUUGCACAAA	CDS	Low	—	31
XM_011511385.3	614-648	*Homo sapiens*	PLEKHB2	AAUGGGCAGGCGUAUGCCGUGCCCUACCAGUACCC	CDS, 3'	Low	—	31
MN339453.1	2961-2995	*Halisarca dujardini*	NAALAD2	AGACAGGGAAGCUUGACAGUGAUCAGGAAGCAUGC	3'	High	—	32
MN339467.1	1869-1903	*Halichondria panicea*	CTH	AGUACCUCAACGGACACAGUGAUGUUGUGAUGGGU	CDS	High	10.1	32
NM_022754.7	997-1031	*Homo sapiens*	SFXN1	AAGCUAAGAUCCAAGAGAGCCAUCCUGAAUUGCGA	CDS	High	—	33
NM_001330400.2	3959-3993	*Homo sapiens*	SFXN5	GGCUGGUGGACAGACCCAGGGUGGUCUUCAAGCCU	3'	High	—	33

Table S3: **Published Ftn IRE-IRP1 Binding Strengths**

	Protein	IRE	Kd (pM)	Method	Reference
1.	human IRP1	human Ftn H	10–30	gel-shift	1
2.	rat IRP1	rat Ftn L	90	gel-shift and filter-binding	2
3.	rabbit IRP1	human Ftn L	20–50	filter-binding	3
4.	human IRP1	human Ftn L	70	gel-shift	4
5.	human IRP1	human Ftn L	14	gel-shift	5
6.	human IRP1	human Ftn L	5–50	gel-shift	6
7.	human IRP1	human Ftn L	50	gel-shift	7
8.	human IRP1	human FtnH	30	competition gel-shift	8
9.	human IRP1	human Ftn L	14	gel-shift	9
10.	rabbit IRP1	frog Ftn L	18	filter-binding	10
11.	rabbit IRP1	frog Ftn L	14000	flourescence quenching	11
12.	rabbit IRP1	frog Ftn L	16000	stopped-flow	12
13.	rabbit IRP1	frog Ftn L	4600–19200	stopped-flow	13

Note

Published Ftn IRE-IRP1 K_d values fall into two groups that differ by three orders of magnitude (1-10: 37. ± 26. pM *vs*. 11-13: 14000. ± 2000. pM). The discrepancies have not been explained.

Table S4. Published PTL Structures With Coordinates Deposited In PDB

PDB ID	Method	Description	Reference
1. 1ANR	NMR	HIV-1 TAR	1
2. 1AQO	NMR	IRE solution structure	2
3. 1ESH	NMR	BMV NMR solution structure	3
4. 1JJ2	XRAY	23S RNA structure	4
5. 1I46, II4B, 1I4C	NMR	BMV NMR solution structure	5
6. 1IK1	NMR	HRV14 RNA hairpin	6
7. 1JZC	NMR	BMV NMR solution structure	7
8. 2IXY, 2IXZ	NMR	HBV ε NMR sol. structure	8
9. 3SNP	XRAY	IRP1:FTH IRE	9
10. 2JTP	NMR	SIV RNA stem-loop; a triloop	10
11. 3SN2	XRAY	IRP1:TFRC B IRE	11
12. 2L5K	NMR	DNA MUC1 23-mer (a triloop)	12
13. 2LP9, 2LPA	NMR	BMV NMR solution structure	13
14. 5J2W	NMR	HIV-1 TAR low-pop intermediate	14
15. 6MCE, 6MCF, 6MCI	NMR	HIV TAT:TAR structures	15

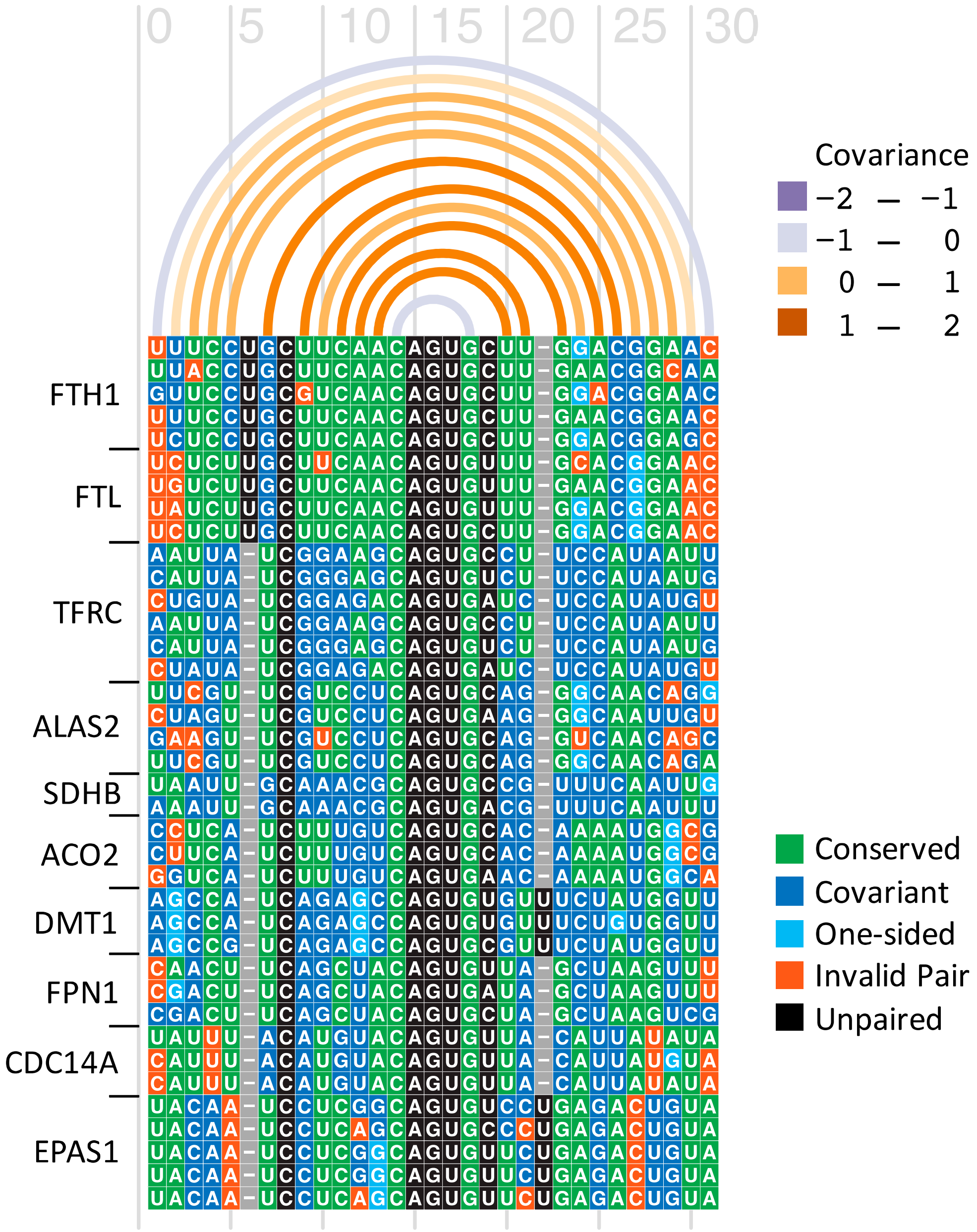

Figure S1. Multiple alignment of the 38 IRE sequences representing the ten IRE-containing genes in Table S1. Display by R-chie/R4RNA [1].

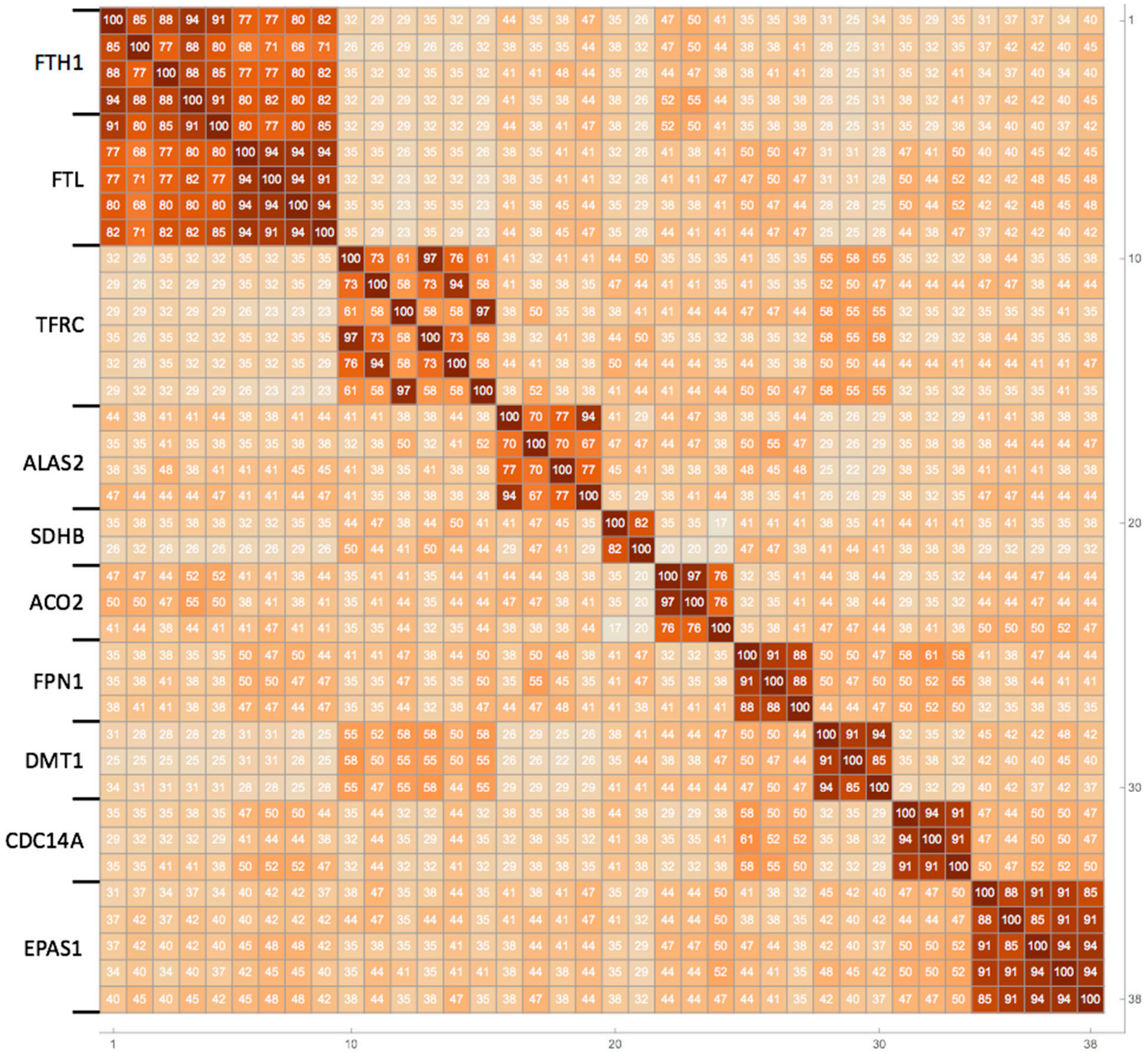

Figure S2. Array of percent identity values for the 38 IRE sequences in Table S1 representing close homologues of the ten IRE-containing genes. While the percent identity per gene type averages 84%, the overall percent identity is 46%. Display by Mathematica [Wolfram Research, Inc., Mathematica, Version 12.1, Champaign, IL (2020)].

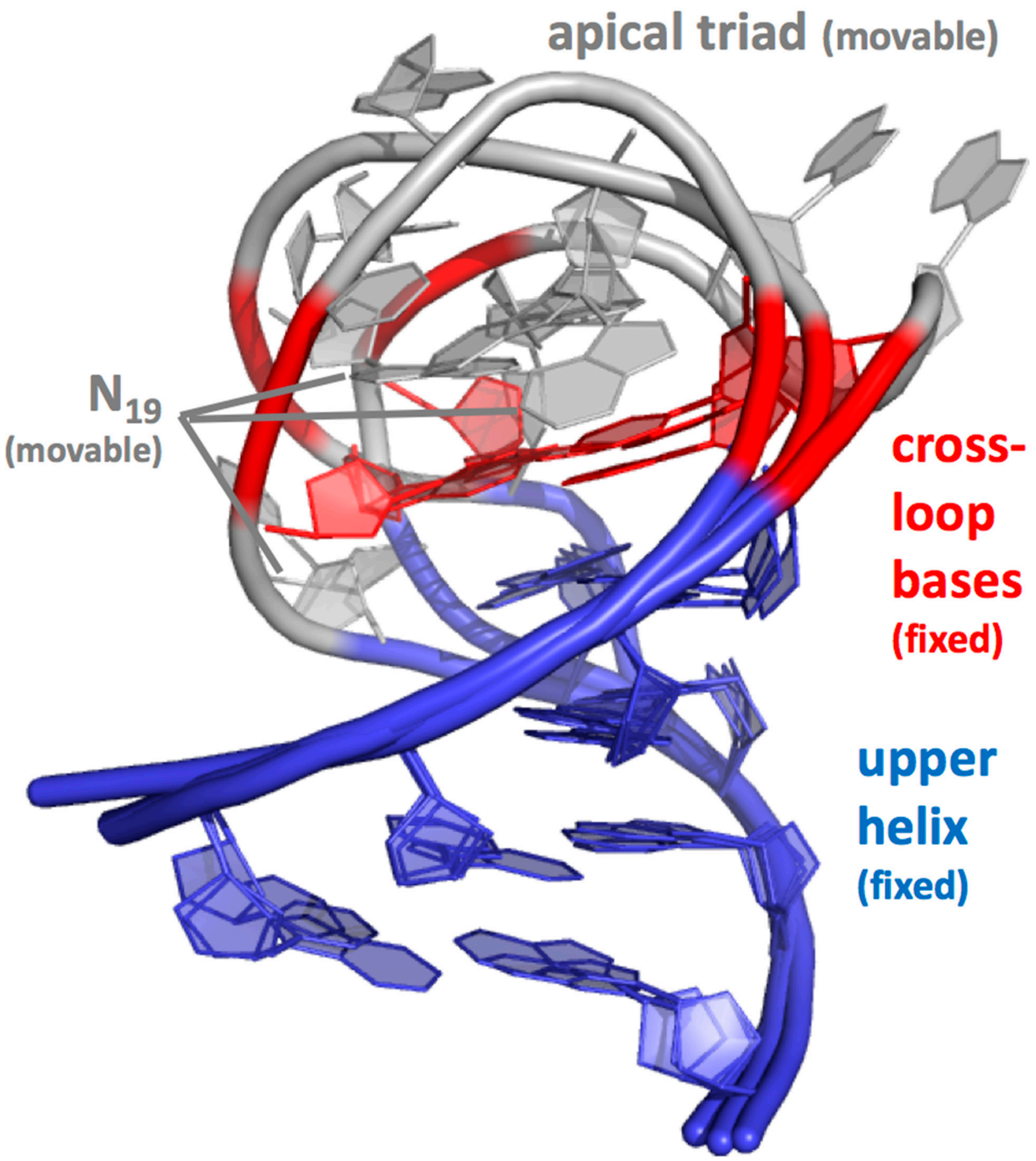

Figure S3. Structural similarities and differences in PTLs from *three unrelated sources*: sub-genomic promoter of BMV (2LP9) [**2**], a 23S rRNA (pdb 1jj2) [**3**], and an FTH1 IRE as bound to IRP1 (3SNP) [**4**]. The three stem-loops were superimposed using only their upper helices (blue). Display by Pymol (DeLano, W. L. The PyMOL User's Manual (2002). DeLano Scientific, San Carlos, CA, USA.).

The purpose of this figure is to contrast the constancy among the helices (blue) and cross-loop base pairs (center, red) with the conformational variability of the apical bases and N_{19}-equivalents (white) among the three completely unrelated PTL structures. The striking structural commonalities are despite the unrelated biological functions and experimental methods used. Note that the bases of the cross-loops are relatively inaccessible [**5**].

While the upper helices and cross-loop bases of the three molecules are positionally the same, the apical triads differ wildly. In published NMR structures of unbound IREs, the recognition bases $A_{15}G_{16}U_{17}$ are disordered and flailing about [**6**, **7**, **8**]. But in the IRE:IRP1 complexes, the recognition bases are ordered and tightly bound to the protein [**4**, **9**, **10**].

The cross-loop is the mainstay of all PTLs, regardless of their function. The important point for the IRE PTL motif is that it is built around the very highly conserved C_{14}-G_{18} cross-loop. This simple rule excludes 25% of the published "IRE-like" sequences (those with positions 14 and/or 18 colored red in Table S2, Column E). Exceptions may exist (*e.g.*, PFN2, [**11**]), but they should be rare.

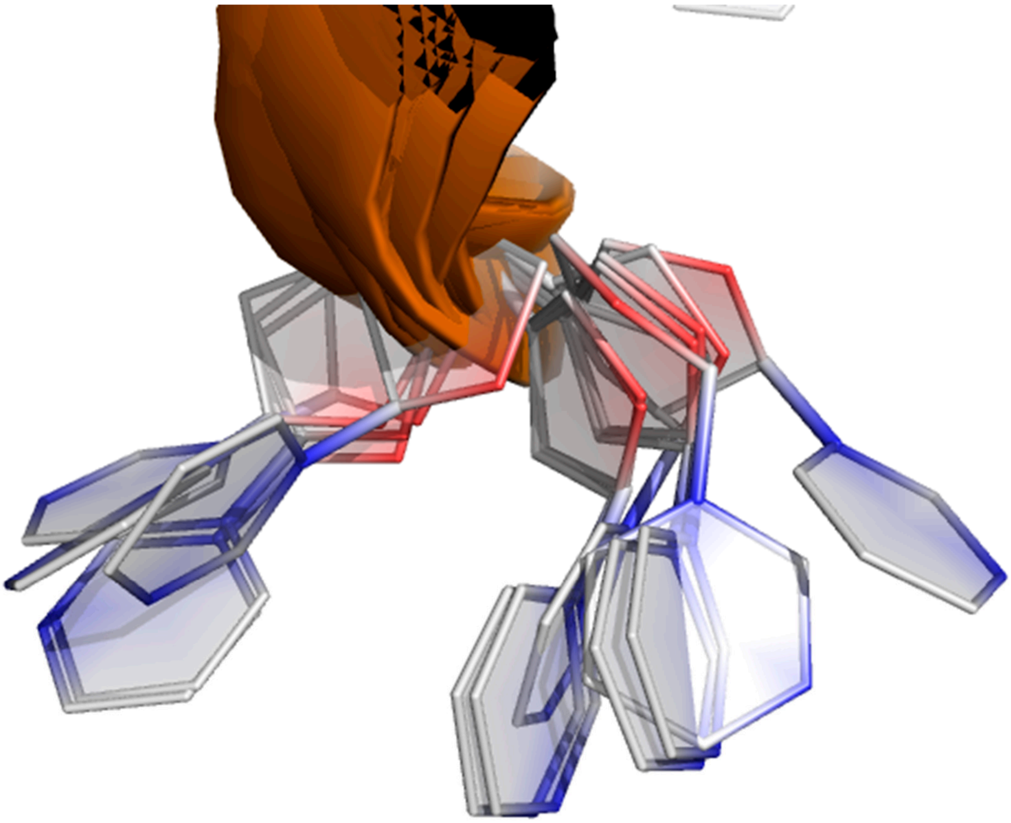

Figure S4. Positions of IRE N_{19} in 12 ocurrences in five x-ray structures of IRE-IRP1 complexes [4, 9, 10]. The figure demonstrates that N_{19} is solvent exposed and has multiple conformations. N_{19} has no protein contacts, and relatively high atomic displacement parameters. The type of base at N_{19} is not functionally important; N_{19} is the PTL place holder. Display by Pymol.

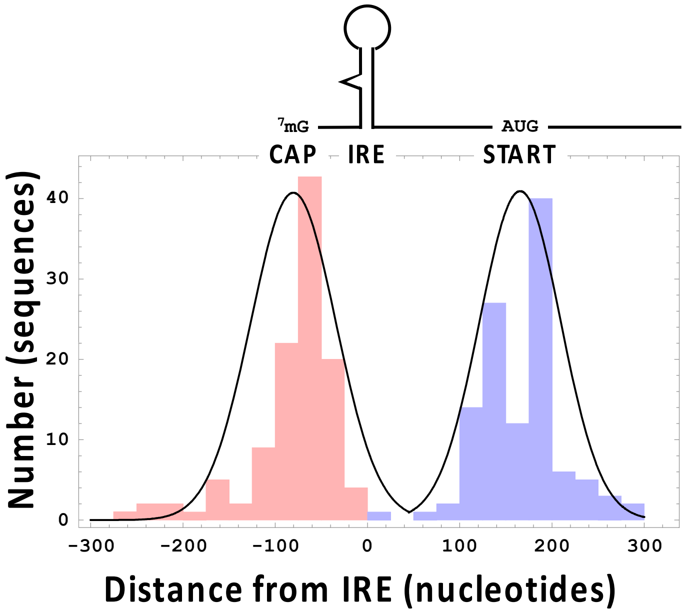

Figure S5. An IRE-centric view of FTH1 5′ UTRs. 110 sequences were used in this example, with an average length of 245 nts. The centers of the IRE stem-loops (nucleotide G_{16}) are at the horizontal axis zero point. The distance distributions from the IREs to the 5′ cap (red) and to the translation start site (blue) have means of -80 and 165 nts respectively (and are uncorrelated). Data were culled from Genbank flatfiles. Unusually long (> 7σ) UTR entries were omitted.

Distances such as these can be additional criteria for screening prospective IRE sequences [12, 13, 14, 15, 16].

Table S1 References

(1) Leibold, E. A.; Munro, H. N. Characterization and Evolution of the Expressed Rat Ferritin Light Subunit Gene and Its Pseudogene Family. Conservation of Sequences within Noncoding Regions of Ferritin Genes. *J Biol Chem* **1987**, *262* (15), 7335–7341.

(2) Hentze, M. W.; Caughman, S. W.; Rouault, T. A.; Barriocanal, J. G.; Dancis, A.; Harford, J. B.; Klausner, R. D. Identification of the Iron-Responsive Element for the Translational Regulation of Human Ferritin mRNA. *Science* **1987**, *238* (4833), 1570–1573. https://doi.org/10.1126/science.3685996.

(3) Aziz, N.; Munro, H. N. Iron Regulates Ferritin mRNA Translation through a Segment of Its 5′ Untranslated Region. *Proc Natl Acad Sci U S A* **1987**, *84* (23), 8478–8482. https://doi.org/10.1073/pnas.84.23.8478.

(4) Leibold, E. A.; Munro, H. N. Cytoplasmic Protein Binds in Vitro to a Highly Conserved Sequence in the 5′ Untranslated Region of Ferritin Heavy- and Light-Subunit mRNAs. *Proc Natl Acad Sci U S A* **1988**, *85* (7), 2171–2175. https://doi.org/10.1073/pnas.85.7.2171.

(5) Walden, W. E.; Daniels-McQueen, S.; Brown, P. H.; Gaffield, L.; Russell, D. A.; Bielser, D.; Bailey, L. C.; Thach, R. E. Translational Repression in Eukaryotes: Partial Purification and Characterization of a Repressor of Ferritin mRNA Translation. *Proc Natl Acad Sci U S A* **1988**, *85* (24), 9503–9507. https://doi.org/10.1073/pnas.85.24.9503.

(6) Koeller, D. M.; Casey, J. L.; Hentze, M. W.; Gerhardt, E. M.; Chan, L. N.; Klausner, R. D.; Harford, J. B. A Cytosolic Protein Binds to Structural Elements within the Iron Regulatory Region of the Transferrin Receptor mRNA. *Proc Natl Acad Sci U S A* **1989**, *86* (10), 3574–3578. https://doi.org/10.1073/pnas.86.10.3574.

(7) Dandekar, T.; Stripecke, R.; Gray, N. K.; Goossen, B.; Constable, A.; Johansson, H. E.; Hentze, M. W. Identification of a Novel Iron-Responsive Element in Murine and Human Erythroid Delta-Aminolevulinic Acid Synthase mRNA. *EMBO J* **1991**, *10* (7), 1903–1909.

(8) Cox, T. C.; Bawden, M. J.; Martin, A.; May, B. K. Human Erythroid 5-Aminolevulinate Synthase: Promoter Analysis and Identification of an Iron-Responsive Element in the mRNA. *EMBO J* **1991**, *10* (7), 1891–1902.

(9) Wingert, R. A.; Galloway, J. L.; Barut, B.; Foott, H.; Fraenkel, P.; Axe, J. L.; Weber, G. J.; Dooley, K.; Davidson, A. J.; Schmid, B.; Schmidt, B.; Paw, B. H.; Shaw, G. C.; Kingsley, P.; Palis, J.; Schubert, H.; Chen, O.; Kaplan, J.; Zon, L. I.; Tübingen 2000 Screen Consortium. Deficiency of Glutaredoxin 5 Reveals Fe-S Clusters Are Required for Vertebrate Haem Synthesis. *Nature* **2005**, *436* (7053), 1035–1039. https://doi.org/10.1038/nature03887.

(10) Kohler, S. A.; Henderson, B. R.; Kühn, L. C. Succinate Dehydrogenase b mRNA of Drosophila Melanogaster Has a Functional Iron-Responsive Element in Its 5′-Untranslated Region. *J Biol Chem* **1995**, *270* (51), 30781–30786. https://doi.org/10.1074/jbc.270.51.30781.

(11) Melefors, O. Translational Regulation in Vivo of the Drosophila Melanogaster mRNA Encoding Succinate Dehydrogenase Iron Protein via Iron Responsive Elements. *Biochem Biophys Res Commun* **1996**, *221* (2), 437–441. https://doi.org/10.1006/bbrc.1996.0613.

(12) Gray, N. K.; Pantopoulos, K.; Dandekar, T.; Ackrell, B. A.; Hentze, M. W. Translational Regulation of Mammalian and Drosophila Citric Acid Cycle Enzymes via Iron-Responsive Elements. *Proc Natl Acad Sci U S A* **1996**, *93* (10), 4925–4930. https://doi.org/10.1073/pnas.93.10.4925.

(13) Kim, H. Y.; LaVaute, T.; Iwai, K.; Klausner, R. D.; Rouault, T. A. Identification of a Conserved and Functional Iron-Responsive Element in the 5′-Untranslated Region of Mammalian Mitochondrial Aconitase. *J Biol Chem* **1996**, *271* (39), 24226–24230. https://doi.org/10.1074/jbc.271.39.24226.

(14) Abboud, S.; Haile, D. J. A Novel Mammalian Iron-Regulated Protein Involved in Intracellular Iron Metabolism. *J Biol Chem* **2000**, *275* (26), 19906–19912. https://doi.org/10.1074/jbc.M000713200.

(15) Donovan, A.; Brownlie, A.; Zhou, Y.; Shepard, J.; Pratt, S. J.; Moynihan, J.; Paw, B. H.; Drejer, A.; Barut, B.; Zapata, A.; Law, T. C.; Brugnara, C.; Lux, S. E.; Pinkus, G. S.; Pinkus, J. L.; Kingsley, P. D.; Palis, J.; Fleming, M. D.; Andrews, N. C.; Zon, L. I. Positional Cloning of Zebrafish Ferroportin1 Identifies a Conserved Vertebrate Iron Exporter. *Nature* **2000**, *403* (6771), 776–781. https://doi.org/10.1038/35001596.

(16) McKie, A. T.; Marciani, P.; Rolfs, A.; Brennan, K.; Wehr, K.; Barrow, D.; Miret, S.; Bomford, A.; Peters, T. J.; Farzaneh, F.; Hediger, M. A.; Hentze, M. W.; Simpson, R. J. A Novel Duodenal Iron-Regulated Transporter, IREG1, Implicated in the Basolateral Transfer of Iron to the Circulation. *Mol Cell* **2000**, *5* (2), 299–309. https://doi.org/10.1016/s1097-2765(00)80425-6.

(17) Lymboussaki, A.; Pignatti, E.; Montosi, G.; Garuti, C.; Haile, D. J.; Pietrangelo, A. The Role of the Iron Responsive Element in the Control of Ferroportin1/IREG1/MTP1 Gene Expression. *J Hepatol* **2003**, *39* (5), 710–715. https://doi.org/10.1016/s0168-8278(03)00408-2.

(18) Gunshin, H.; Allerson, C. R.; Polycarpou-Schwarz, M.; Rofts, A.; Rogers, J. T.; Kishi, F.; Hentze, M. W.; Rouault, T. A.; Andrews, N. C.; Hediger, M. A. Iron-Dependent Regulation of the Divalent Metal Ion Transporter. *FEBS Lett* **2001**, *509* (2), 309–316. https://doi.org/10.1016/s0014-5793(01)03189-1.

(19) Sanchez, M.; Galy, B.; Dandekar, T.; Bengert, P.; Vainshtein, Y.; Stolte, J.; Muckenthaler, M. U.; Hentze, M. W. Iron Regulation and the Cell Cycle: Identification of an Iron-Responsive Element in the 3′-Untranslated Region of Human Cell Division Cycle 14A mRNA by a Refined Microarray-Based Screening Strategy. *J Biol Chem* **2006**, *281* (32), 22865–22874. https://doi.org/10.1074/jbc.M603876200.

(20) Sanchez, M.; Galy, B.; Muckenthaler, M. U.; Hentze, M. W. Iron-Regulatory Proteins Limit Hypoxia-Inducible Factor-2α Expression in Iron Deficiency. *Nat Struct Mol Biol* **2007**, *14* (5), 420–426. https://doi.org/10.1038/nsmb1222.

Table S2 References

(1) Cox, L. A.; Adrian, G. S. Posttranscriptional Regulation of Chimeric Human Transferrin Genes by Iron. *Biochemistry* **1993**, *32* (18), 4738–4745. https://doi.org/10.1021/bi00069a007.

(2) Lim, K. C.; Ishihara, H.; Riddle, R. D.; Yang, Z.; Andrews, N.; Yamamoto, M.; Engel, J. D. Structure and Regulation of the Chicken Erythroid δ-Aminolevulinate Synthase Gene. *Nucleic Acids Res* **1994**, *22* (7), 1226–1233. https://doi.org/10.1093/nar/22.7.1226.

(3) Leedman, P. J.; Stein, A. R.; Chin, W. W.; Rogers, J. T. Thyroid Hormone Modulates the Interaction between Iron Regulatory Proteins and the Ferritin mRNA Iron-Responsive Element. *J Biol Chem* **1996**, *271* (20), 12017–12023. https://doi.org/10.1074/jbc.271.20.12017.

(4) Dandekar, T.; Beyer, K.; Bork, P.; Kenealy, M. R.; Pantopoulos, K.; Hentze, M.; Sonntag-Buck, V.; Flouriot, G.; Gannon, F.; Schreiber, S. Systematic Genomic Screening and Analysis of mRNA in Untranslated Regions and mRNA Precursors: Combining Experimental and Computational Approaches. *Bioinformatics* **1998**, *14* (3), 271–278. https://doi.org/10.1093/bioinformatics/14.3.271.

(5) Lin, E.; Graziano, J. H.; Freyer, G. A. Regulation of the 75-KDa Subunit of Mitochondrial Complex I by Iron. *J Biol Chem* **2001**, *276* (29), 27685–27692. https://doi.org/10.1074/jbc.M100941200.

(6) Kohler, S. A.; Menotti, E.; Kühn, L. C. Molecular Cloning of Mouse Glycolate Oxidase. High Evolutionary Conservation and Presence of an Iron-Responsive Element-like Sequence in the mRNA. *J Biol Chem* **1999**, *274* (4), 2401–2407. https://doi.org/10.1074/jbc.274.4.2401.

(7) Macke, T. J.; Ecker, D. J.; Gutell, R. R.; Gautheret, D.; Case, D. A.; Sampath, R. RNAMotif, an RNA Secondary Structure Definition and Search Algorithm. *Nucleic Acids Res* **2001**, *29* (22), 4724–4735. https://doi.org/10.1093/nar/29.22.4724.

(8) Stevens, S. G.; Gardner, P. P.; Brown, C. Two Covariance Models for Iron-Responsive Elements. *RNA Biol* **2011**, *8* (5), 792–801. https://doi.org/10.4161/rna.8.5.16037.
(9) Rogers, J. T.; Randall, J. D.; Cahill, C. M.; Eder, P. S.; Huang, X.; Gunshin, H.; Leiter, L.; McPhee, J.; Sarang, S. S.; Utsuki, T.; Greig, N. H.; Lahiri, D. K.; Tanzi, R. E.; Bush, A. I.; Giordano, T.; Gullans, S. R. An Iron-Responsive Element Type II in the 5′-Untranslated Region of the Alzheimer's Amyloid Precursor Protein Transcript. *J Biol Chem* **2002**, *277* (47), 45518–45528. https://doi.org/10.1074/jbc.M207435200.
(10) Loyevsky, M.; Mompoint, F.; Yikilmaz, E.; Altschul, S. F.; Madden, T.; Wootton, J. C.; Kurantsin-Mills, J.; Kassim, O. O.; Gordeuk, V. R.; Rouault, T. A. Expression of a Recombinant IRP-like Plasmodium Falciparum Protein That Specifically Binds Putative Plasmodial IREs. *Mol Biochem Parasitol* **2003**, *126* (2), 231–238. https://doi.org/10.1016/s0166-6851(02)00278-5.
(11) Huang, T. S.; Law, J. H.; Söderhäll, K. Purification and CDNA Cloning of Ferritin from the Hepatopancreas of the Freshwater Crayfish *Pacifastacus Leniusculus*. *Eur J Biochem* **1996**, *236* (2), 450–456. https://doi.org/10.1111/j.1432-1033.1996.00450.x.
(12) Hsieh, S.-L.; Chiu, Y.-C.; Kuo, C.-M. Molecular Cloning and Tissue Distribution of Ferritin in Pacific White Shrimp (*Litopenaeus Vannamei*). *Fish Shellfish Immunol* **2006**, *21* (3), 279–283. https://doi.org/10.1016/j.fsi.2005.12.003.
(13) Jeong, B. R.; Chung, S.-M.; Baek, N. J.; Koo, K. B.; Baik, H. S.; Joo, H.-S.; Chang, C.-S.; Choi, J. W. Characterization, Cloning and Expression of the Ferritin Gene from the Korean Polychaete, *Periserrula Leucophryna*. *J Microbiol* **2006**, *44* (1), 54–63.
(14) Cmejla, R.; Petrak, J.; Cmejlova, J. A Novel Iron Responsive Element in the 3'UTR of Human MRCKα. *Biochem Biophys Res Commun* **2006**, *341* (1), 158–166. https://doi.org/10.1016/j.bbrc.2005.12.155.
(15) Sanchez, M.; Galy, B.; Dandekar, T.; Bengert, P.; Vainshtein, Y.; Stolte, J.; Muckenthaler, M. U.; Hentze, M. W. Iron Regulation and the Cell Cycle: Identification of an Iron-Responsive Element in the 3′-Untranslated Region of Human Cell Division Cycle 14A mRNA by a Refined Microarray-Based Screening Strategy. *J Biol Chem* **2006**, *281* (32), 22865–22874. https://doi.org/10.1074/jbc.M603876200.
(16) Friedlich, A. L.; Tanzi, R. E.; Rogers, J. T. The 5′-Untranslated Region of Parkinson's Disease α-Synuclein MessengerRNA Contains a Predicted Iron Responsive Element. *Mol Psychiatry* **2007**, *12* (3), 222–223. https://doi.org/10.1038/sj.mp.4001937.
(17) Rogers, J. T.; Mikkilineni, S.; Cantuti-Castelvetri, I.; Smith, D. H.; Huang, X.; Bandyopadhyay, S.; Cahill, C. M.; Maccecchini, M. L.; Lahiri, D. K.; Greig, N. H. The α Synuclein 5′untranslated Region Targeted Translation Blockers: Anti- α Synuclein Efficacy of Cardiac Glycosides and Posiphen. *J Neural Transm (Vienna)* **2011**, *118* (3), 493–507. https://doi.org/10.1007/s00702-010-0513-5.
(18) Mikkilineni, S.; Cantuti-Castelvetri, I.; Cahill, C. M.; Balliedier, A.; Greig, N. H.; Rogers, J. T. The Anticholinesterase Phenserine and Its Enantiomer Posiphen as 5′ Untranslated-Region-Directed Translation Blockers of the Parkinson's Alpha Synuclein Expression. *Parkinsons Dis* **2012**, *2012*, 142372. https://doi.org/10.1155/2012/142372.
(19) Banerjee, S.; Nandyala, A. K.; Raviprasad, P.; Ahmed, N.; Hasnain, S. E. Iron-Dependent RNA-Binding Activity of Mycobacterium Tuberculosis Aconitase. *J Bacteriol* **2007**, *189* (11), 4046–4052. https://doi.org/10.1128/JB.00026-07.
(20) Solano-González, E.; Burrola-Barraza, E.; León-Sicairos, C.; Avila-González, L.; Gutiérrez-Escolano, L.; Ortega-López, J.; Arroyo, R. The Trichomonad Cysteine Proteinase TVCP4 Transcript Contains an Iron-Responsive Element. *FEBS Lett* **2007**, *581* (16), 2919–2928. https://doi.org/10.1016/j.febslet.2007.05.056.

(21) Figueroa-Angulo, E. E.; Calla-Choque, J. S.; Mancilla-Olea, M. I.; Arroyo, R. RNA-Binding Proteins in *Trichomonas Vaginalis*: Atypical Multifunctional Proteins. *Biomolecules* **2015**, *5* (4), 3354–3395. https://doi.org/10.3390/biom5043354.

(22) dos Santos, C. O.; Dore, L. C.; Valentine, E.; Shelat, S. G.; Hardison, R. C.; Ghosh, M.; Wang, W.; Eisenstein, R. S.; Costa, F. F.; Weiss, M. J. An Iron Responsive Element-like Stem-Loop Regulates α -Hemoglobin-Stabilizing Protein mRNA. *J Biol Chem* **2008**, *283* (40), 26956–26964. https://doi.org/10.1074/jbc.M802421200.

(23) Ande, S. R.; Mishra, S. Nuclear Coded Mitochondrial Protein Prohibitin Is an Iron Regulated Iron Binding Protein. *Mitochondrion* **2011**, *11* (1), 40–47. https://doi.org/10.1016/j.mito.2010.07.002.

(24) Sanchez, M.; Galy, B.; Schwanhaeusser, B.; Blake, J.; Bähr-Ivacevic, T.; Benes, V.; Selbach, M.; Muckenthaler, M. U.; Hentze, M. W. Iron Regulatory Protein-1 and -2: Transcriptome-Wide Definition of Binding mRNAs and Shaping of the Cellular Proteome by Iron Regulatory Proteins. *Blood* **2011**, *118* (22), e168-179. https://doi.org/10.1182/blood-2011-04-343541.

(25) Livesey, J. A.; Manning, R. A.; Meek, J. H.; Jackson, J. E.; Kulinskaya, E.; Laffan, M. A.; Shovlin, C. L. Low Serum Iron Levels Are Associated with Elevated Plasma Levels of Coagulation Factor VIII and Pulmonary Emboli/Deep Venous Thromboses in Replicate Cohorts of Patients with Hereditary Haemorrhagic Telangiectasia. *Thorax* **2012**, *67* (4), 328–333. https://doi.org/10.1136/thoraxjnl-2011-201076.

(26) Liu, Z.; Lanford, R.; Mueller, S.; Gerhard, G. S.; Luscieti, S.; Sanchez, M.; Devireddy, L. Siderophore-Mediated Iron Trafficking in Humans Is Regulated by Iron. *J Mol Med (Berl)* **2012**, *90* (10), 1209–1221. https://doi.org/10.1007/s00109-012-0899-7.

(27) Luscieti, S.; Galy, B.; Gutierrez, L.; Reinke, M.; Couso, J.; Shvartsman, M.; Di Pascale, A.; Witke, W.; Hentze, M. W.; Pilo Boyl, P.; Sanchez, M. The Actin-Binding Protein Profilin 2 Is a Novel Regulator of Iron Homeostasis. *Blood* **2017**, *130* (17), 1934–1945. https://doi.org/10.1182/blood-2016-11-754382.

(28) Liang, H.; Huang, H.; Tan, P.-Z.; Liu, Y.; Nie, J.-H.; Zhang, Y.-T.; Zhang, K.-L.; Diao, Y.; He, Q.; Hou, B.-Y.; Zhao, T.-T.; Li, Y.-Z.; Lv, G.-X.; Lee, K.-Y.; Gao, X.; Zhou, L.-Y. Effect of Iron on Cholesterol 7α-Hydroxylase Expression in Alcohol-Induced Hepatic Steatosis in Mice. *J Lipid Res* **2017**, *58* (8), 1548–1560. https://doi.org/10.1194/jlr.M074534.

(29) Lu, M. A.; Rajanala, S.; Mikkilineni, S. V.; Cahill, C. M.; Brown, R.; Berry, J. D.; Rogers, J. T. The 5′-Untranslated Region of the C9orf72 mRNA Exhibits a Phylogenetic Alignment to the *cis*-Aconitase Iron-Responsive Element; Novel Therapies for Amytrophic Lateral Sclerosis. *Neuroscience & Medicine* **2016**, *7*, 15–26.

(30) Soto-Castro, L.; Plata-Guzmán, L. Y.; Figueroa-Angulo, E. E.; Calla-Choque, J. S.; Reyes-López, M.; de la Garza, M.; León-Sicairos, N.; Garzón-Tiznado, J. A.; Arroyo, R.; León-Sicairos, C. Iron Responsive-like Elements in the Parasite *Entamoeba Histolytica*. *Microbiology (Reading)* **2017**, *163* (9), 1329–1342. https://doi.org/10.1099/mic.0.000431.

(31) Connell, G. J.; Danial, J. S.; Haastruthers, C. X. Evaluation of the Iron Regulatory Protein-1 Interactome. *Biometals* **2018**, *31* (1), 139–146. https://doi.org/10.1007/s10534-018-0076-8.

(32) Finoshin, A. D.; Adameyko, K. I.; Mikhailov, K. V.; Kravchuk, O. I.; Georgiev, A. A.; Gornostaev, N. G.; Kosevich, I. A.; Mikhailov, V. S.; Gazizova, G. R.; Shagimardanova, E. I.; Gusev, O. A.; Lyupina, Y. V. Iron Metabolic Pathways in the Processes of Sponge Plasticity. *PLoS One* **2020**, *15* (2), e0228722. https://doi.org/10.1371/journal.pone.0228722.

(33) Tifoun, N.; De Las Heras, J. M.; Guillaume, A.; Bouleau, S.; Mignotte, B.; Le Floch, N. Insights into the Roles of the Sideroflexins/SLC56 Family in Iron Homeostasis and Iron-Sulfur Biogenesis. *Biomedicines* **2021**, *9* (2), 103. https://doi.org/10.3390/biomedicines9020103.

Table S3 References

(1) Haile, D. J.; Hentze, M. W.; Rouault, T. A.; Harford, J. B.; Klausner, R. D. Regulation of Interaction of the Iron-Responsive Element Binding Protein with Iron-Responsive RNA Elements. *Mol Cell Biol* **1989**, *9* (11), 5055–5061. https://doi.org/10.1128/mcb.9.11.5055-5061.1989.

(2) Barton, H. A.; Eisenstein, R. S.; Bomford, A.; Munro, H. N. Determinants of the Interaction between the Iron-Responsive Element-Binding Protein and Its Binding Site in Rat L-Ferritin mRNA. *J Biol Chem* **1990**, *265* (12), 7000–7008.

(3) Swenson, G. R.; Patino, M. M.; Beck, M. M.; Gaffield, L.; Walden, W. E. Characteristics of the Interaction of the Ferritin Repressor Protein with the Iron-Responsive Element. *Biol Met* **1991**, *4* (1), 48–55. https://doi.org/10.1007/BF01135557.

(4) Kaptain, S.; Downey, W. E.; Tang, C.; Philpott, C.; Haile, D.; Orloff, D. G.; Harford, J. B.; Rouault, T. A.; Klausner, R. D. A Regulated RNA Binding Protein Also Possesses Aconitase Activity. *Proc Natl Acad Sci U S A* **1991**, *88* (22), 10109–10113. https://doi.org/10.1073/pnas.88.22.10109.

(5) Philpott, C. C.; Klausner, R. D.; Rouault, T. A. The Bifunctional Iron-Responsive Element Binding Protein/Cytosolic Aconitase: The Role of Active-Site Residues in Ligand Binding and Regulation. *Proc Natl Acad Sci U S A* **1994**, *91* (15), 7321–7325. https://doi.org/10.1073/pnas.91.15.7321.

(6) Butt, J.; Kim, H. Y.; Basilion, J. P.; Cohen, S.; Iwai, K.; Philpott, C. C.; Altschul, S.; Klausner, R. D.; Rouault, T. A. Differences in the RNA Binding Sites of Iron Regulatory Proteins and Potential Target Diversity. *Proc Natl Acad Sci U S A* **1996**, *93* (9), 4345–4349. https://doi.org/10.1073/pnas.93.9.4345.

(7) Allerson, C. R.; Cazzola, M.; Rouault, T. A. Clinical Severity and Thermodynamic Effects of Iron-Responsive Element Mutations in Hereditary Hyperferritinemia-Cataract Syndrome. *J Biol Chem* **1999**, *274* (37), 26439–26447. https://doi.org/10.1074/jbc.274.37.26439.

(8) Gunshin, H.; Allerson, C. R.; Polycarpou-Schwarz, M.; Rofts, A.; Rogers, J. T.; Kishi, F.; Hentze, M. W.; Rouault, T. A.; Andrews, N. C.; Hediger, M. A. Iron-Dependent Regulation of the Divalent Metal Ion Transporter. *FEBS Lett* **2001**, *509* (2), 309–316. https://doi.org/10.1016/s0014-5793(01)03189-1.

(9) Goforth, J. B.; Anderson, S. A.; Nizzi, C. P.; Eisenstein, R. S. Multiple Determinants within Iron-Responsive Elements Dictate Iron Regulatory Protein Binding and Regulatory Hierarchy. *RNA* **2010**, *16* (1), 154–169. https://doi.org/10.1261/rna.1857210.

(10) Selezneva, A. I.; Walden, W. E.; Volz, K. W. Nucleotide-Specific Recognition of Iron-Responsive Elements by Iron Regulatory Protein 1. *J Mol Biol* **2013**, *425* (18), 3301–3310. https://doi.org/10.1016/j.jmb.2013.06.023.

(11) Khan, M. A.; Walden, W. E.; Goss, D. J.; Theil, E. C. Direct Fe2+ Sensing by Iron-Responsive Messenger RNA:Repressor Complexes Weakens Binding. *J Biol Chem* **2009**, *284* (44), 30122–30128. https://doi.org/10.1074/jbc.M109.041061.

(12) Khan, M. A.; Ma, J.; Walden, W. E.; Merrick, W. C.; Theil, E. C.; Goss, D. J. Rapid Kinetics of Iron Responsive Element (IRE) RNA/Iron Regulatory Protein 1 and IRE-RNA/EIF4F Complexes Respond Differently to Metal Ions. *Nucleic Acids Res* **2014**, *42* (10), 6567–6577. https://doi.org/10.1093/nar/gku248.

(13) Khan, M. A.; Walden, W. E.; Theil, E. C.; Goss, D. J. Thermodynamic and Kinetic Analyses of Iron Response Element (IRE)-mRNA Binding to Iron Regulatory Protein, IRP1. *Sci Rep* **2017**, *7* (1), 8532. https://doi.org/10.1038/s41598-017-09093-5.

Table S4 References

(1) Aboul-ela, F.; Karn, J.; Varani, G. Structure of HIV-1 TAR RNA in the Absence of Ligands Reveals a Novel Conformation of the Trinucleotide Bulge. *Nucleic Acids Res* **1996**, *24* (20), 3974–3981. https://doi.org/10.1093/nar/24.20.3974.

(2) Addess, K. J.; Basilion, J. P.; Klausner, R. D.; Rouault, T. A.; Pardi, A. Structure and Dynamics of the Iron Responsive Element RNA: Implications for Binding of the RNA by Iron Regulatory Binding Proteins. *J Mol Biol* **1997**, *274* (1), 72–83. https://doi.org/10.1006/jmbi.1997.1377.

(3) Kim, C. H.; Kao, C. C.; Tinoco, I. RNA Motifs That Determine Specificity between a Viral Replicase and Its Promoter. *Nat Struct Biol* **2000**, *7* (5), 415–423. https://doi.org/10.1038/75202.

(4) Klein, D. J.; Schmeing, T. M.; Moore, P. B.; Steitz, T. A. The Kink-Turn: A New RNA Secondary Structure Motif. *EMBO J* **2001**, *20* (15), 4214–4221. https://doi.org/10.1093/emboj/20.15.4214.

(5) Kim, C. H.; Tinoco, I. Structural and Thermodynamic Studies on Mutant RNA Motifs That Impair the Specificity between a Viral Replicase and Its Promoter. *J Mol Biol* **2001**, *307* (3), 827–839. https://doi.org/10.1006/jmbi.2001.4497.

(6) Huang, H.; Alexandrov, A.; Chen, X.; Barnes, T. W.; Zhang, H.; Dutta, K.; Pascal, S. M. Structure of an RNA Hairpin from HRV-14. *Biochemistry* **2001**, *40* (27), 8055–8064. https://doi.org/10.1021/bi010572b.

(7) Kim, C. H.; Kao, C. C. A Mutant Viral RNA Promoter with an Altered Conformation Retains Efficient Recognition by a Viral RNA Replicase through a Solution-Exposed Adenine. *RNA* **2001**, *7* (10), 1476–1485.

(8) Flodell, S.; Petersen, M.; Girard, F.; Zdunek, J.; Kidd-Ljunggren, K.; Schleucher, J.; Wijmenga, S. Solution Structure of the Apical Stem-Loop of the Human Hepatitis B Virus Encapsidation Signal. *Nucleic Acids Res* **2006**, *34* (16), 4449–4457. https://doi.org/10.1093/nar/gkl582.

(9) Walden, W. E.; Selezneva, A. I.; Dupuy, J.; Volbeda, A.; Fontecilla-Camps, J. C.; Theil, E. C.; Volz, K. Structure of Dual Function Iron Regulatory Protein 1 Complexed with Ferritin IRE-RNA. *Science* **2006**, *314* (5807), 1903–1908. https://doi.org/10.1126/science.1133116.

(10) Marcheschi, R. J.; Staple, D. W.; Butcher, S. E. Programmed Ribosomal Frameshifting in SIV Is Induced by a Highly Structured RNA Stem-Loop. *J Mol Biol* **2007**, *373* (3), 652–663. https://doi.org/10.1016/j.jmb.2007.08.033.

(11) Walden, W. E.; Selezneva, A.; Volz, K. Accommodating Variety in Iron-Responsive Elements: Crystal Structure of Transferrin Receptor 1 B IRE Bound to Iron Regulatory Protein 1. *FEBS Lett* **2012**, *586* (1), 32–35. https://doi.org/10.1016/j.febslet.2011.11.018.

(12) Baouendi, M.; Cognet, J. A. H.; Ferreira, C. S. M.; Missailidis, S.; Coutant, J.; Piotto, M.; Hantz, E.; Hervé du Penhoat, C. Solution Structure of a Truncated Anti-MUC1 DNA Aptamer Determined by Mesoscale Modeling and NMR. *FEBS J* **2012**, *279* (3), 479–490. https://doi.org/10.1111/j.1742-4658.2011.08440.x.

(13) Skov, J.; Gaudin, M.; Podbevsek, P.; Olsthoorn, R. C. L.; Petersen, M. The Subgenomic Promoter of Brome Mosaic Virus Folds into a Stem-Loop Structure Capped by a Pseudo-Triloop That Is Structurally Similar to the Triloop of the Genomic Promoter. *RNA* **2012**, *18* (5), 992–1000. https://doi.org/10.1261/rna.029918.111.

(14) Borkar, A. N.; Bardaro, M. F.; Camilloni, C.; Aprile, F. A.; Varani, G.; Vendruscolo, M. Structure of a Low-Population Binding Intermediate in Protein-RNA Recognition. *Proc Natl Acad Sci U S A* **2016**, *113* (26), 7171–7176. https://doi.org/10.1073/pnas.1521349113.

(15) Pham, V. V.; Salguero, C.; Khan, S. N.; Meagher, J. L.; Brown, W. C.; Humbert, N.; de Rocquigny, H.; Smith, J. L.; D'Souza, V. M. HIV-1 Tat Interactions with Cellular 7SK and Viral TAR RNAs Identifies Dual Structural Mimicry. *Nat Commun* **2018**, *9* (1), 4266. https://doi.org/10.1038/s41467-018-06591-6.

Figures S1-S5 References

(1) Lai, D.; Proctor, J. R.; Zhu, J. Y. A.; Meyer, I. M. R-CHIE: A Web Server and R Package for Visualizing RNA Secondary Structures. *Nucleic Acids Res* **2012**, *40* (12), e95. https://doi.org/10.1093/nar/gks241.

(2) Skov, J.; Gaudin, M.; Podbevsek, P.; Olsthoorn, R. C. L.; Petersen, M. The Subgenomic Promoter of Brome Mosaic Virus Folds into a Stem-Loop Structure Capped by a Pseudo-Triloop That Is Structurally Similar to the Triloop of the Genomic Promoter. *RNA* **2012**, *18* (5), 992–1000. https://doi.org/10.1261/rna.029918.111.

(3) Klein, D. J.; Schmeing, T. M.; Moore, P. B.; Steitz, T. A. The Kink-Turn: A New RNA Secondary Structure Motif. *EMBO J* **2001**, *20* (15), 4214–4221. https://doi.org/10.1093/emboj/20.15.4214.

(4) Walden, W. E.; Selezneva, A. I.; Dupuy, J.; Volbeda, A.; Fontecilla-Camps, J. C.; Theil, E. C.; Volz, K. Structure of Dual Function Iron Regulatory Protein 1 Complexed with Ferritin IRE-RNA. *Science* **2006**, *314* (5807), 1903–1908. https://doi.org/10.1126/science.1133116.

(5) Bettany, A. J.; Eisenstein, R. S.; Munro, H. N. Mutagenesis of the Iron-Regulatory Element Further Defines a Role for RNA Secondary Structure in the Regulation of Ferritin and Transferrin Receptor Expression. *J Biol Chem* **1992**, *267* (23), 16531–16537.

(6) Laing, L. G.; Hall, K. B. A Model of the Iron Responsive Element RNA Hairpin Loop Structure Determined from NMR and Thermodynamic Data. *Biochemistry* **1996**, *35* (42), 13586–13596. https://doi.org/10.1021/bi961310q.

(7) Addess, K. J.; Basilion, J. P.; Klausner, R. D.; Rouault, T. A.; Pardi, A. Structure and Dynamics of the Iron Responsive Element RNA: Implications for Binding of the RNA by Iron Regulatory Binding Proteins. *J Mol Biol* **1997**, *274* (1), 72–83. https://doi.org/10.1006/jmbi.1997.1377.

(8) Showalter, S. A.; Baker, N. A.; Tang, C.; Hall, K. B. Iron Responsive Element RNA Flexibility Described by NMR and Isotropic Reorientational Eigenmode Dynamics. *J Biomol NMR* **2005**, *32* (3), 179–193. https://doi.org/10.1007/s10858-005-7948-2.

(9) Walden, W. E.; Selezneva, A.; Volz, K. Accommodating Variety in Iron-Responsive Elements: Crystal Structure of Transferrin Receptor 1 B IRE Bound to Iron Regulatory Protein 1. *FEBS Lett* **2012**, *586* (1), 32–35. https://doi.org/10.1016/j.febslet.2011.11.018.

(10) Selezneva, A. I.; Walden, W. E.; Volz, K. W. Nucleotide-Specific Recognition of Iron-Responsive Elements by Iron Regulatory Protein 1. *J Mol Biol* **2013**, *425* (18), 3301–3310. https://doi.org/10.1016/j.jmb.2013.06.023.

(11) Luscieti, S.; Galy, B.; Gutierrez, L.; Reinke, M.; Couso, J.; Shvartsman, M.; Di Pascale, A.; Witke, W.; Hentze, M. W.; Pilo Boyl, P.; Sanchez, M. The Actin-Binding Protein Profilin 2 Is a Novel Regulator of Iron Homeostasis. *Blood* **2017**, *130* (17), 1934–1945. https://doi.org/10.1182/blood-2016-11-754382.

(12) Goossen, B.; Hentze, M. W. Position Is the Critical Determinant for Function of Iron-Responsive Elements as Translational Regulators. *Mol Cell Biol* **1992**, *12* (5), 1959–1966. https://doi.org/10.1128/mcb.12.5.1959-1966.1992.

(13) Stripecke, R.; Oliveira, C. C.; McCarthy, J. E.; Hentze, M. W. Proteins Binding to 5′ Untranslated Region Sites: A General Mechanism for Translational Regulation of MRNAs in Human and Yeast Cells. *Mol Cell Biol* **1994**, *14* (9), 5898–5909. https://doi.org/10.1128/mcb.14.9.5898-5909.1994.

(14) Paraskeva, E.; Gray, N. K.; Schläger, B.; Wehr, K.; Hentze, M. W. Ribosomal Pausing and Scanning Arrest as Mechanisms of Translational Regulation from Cap-Distal Iron-Responsive Elements. *Mol Cell Biol* **1999**, *19* (1), 807–816. https://doi.org/10.1128/MCB.19.1.807.

(15) Sanchez, M.; Galy, B.; Dandekar, T.; Bengert, P.; Vainshtein, Y.; Stolte, J.; Muckenthaler, M. U.; Hentze, M. W. Iron Regulation and the Cell Cycle: Identification of an Iron-Responsive Element in the 3′-Untranslated Region of Human Cell Division Cycle 14A MRNA by a Refined Microarray-

Based Screening Strategy. *J Biol Chem* **2006**, *281* (32), 22865–22874. https://doi.org/10.1074/jbc.M603876200.

(16) Sanchez, M.; Galy, B.; Muckenthaler, M. U.; Hentze, M. W. Iron-Regulatory Proteins Limit Hypoxia-Inducible Factor-2alpha Expression in Iron Deficiency. *Nat Struct Mol Biol* **2007**, *14* (5), 420–426. https://doi.org/10.1038/nsmb1222.

Script 1: Unix Script for Retrieval of Sequences in Table S1 Using NCBI Utilities

```
esearch -db nuccore -query "NM_002032.3"    | efetch -seq_start  29    -seq_stop 63    -format fasta > NM_002032.3.fa
esearch -db nuccore -query "NM_131585.1"    | efetch -seq_start  3     -seq_stop 37    -format fasta > NM_131585.1.fa
esearch -db nuccore -query "NM_205086.1"    | efetch -seq_start  29    -seq_stop 63    -format fasta > NM_205086.1.fa
esearch -db nuccore -query "NM_010239.2"    | efetch -seq_start  83    -seq_stop 117   -format fasta > NM_010239.2.fa
esearch -db nuccore -query "NM_001172847.1" | efetch -seq_start  2     -seq_stop 36    -format fasta > NM_001172847.1.fa
esearch -db nuccore -query "NM_000146.4"    | efetch -seq_start  23    -seq_stop 57    -format fasta > NM_000146.4.fa
esearch -db nuccore -query "NM_010240.2"    | efetch -seq_start  76    -seq_stop 110   -format fasta > NM_010240.2.fa
esearch -db nuccore -query "NM_022500.4"    | efetch -seq_start  28    -seq_stop 62    -format fasta > NM_022500.4.fa
esearch -db nuccore -query "NM_174792.4"    | efetch -seq_start  79    -seq_stop 113   -format fasta > NM_174792.4.fa
esearch -db nuccore -query "NM_001128148.3" | efetch -seq_start  3286  -seq_stop 3319  -format fasta > NM_001128148.3a.fa
esearch -db nuccore -query "NM_001128148.3" | efetch -seq_start  3690  -seq_stop 3723  -format fasta > NM_001128148.3b.fa
esearch -db nuccore -query "NM_001128148.3" | efetch -seq_start  3755  -seq_stop 3788  -format fasta > NM_001128148.3c.fa
esearch -db nuccore -query "NM_011638.4"    | efetch -seq_start  3336  -seq_stop 3369  -format fasta > NM_011638.4a.fa
esearch -db nuccore -query "NM_011638.4"    | efetch -seq_start  3715  -seq_stop 3748  -format fasta > NM_011638.4b.fa
esearch -db nuccore -query "NM_011638.4"    | efetch -seq_start  3780  -seq_stop 3813  -format fasta > NM_011638.4c.fa
esearch -db nuccore -query "NM_000032.5"    | efetch -seq_start  6     -seq_stop 39    -format fasta > NM_000032.5.fa
esearch -db nuccore -query "XM_002125731.4" | efetch -seq_start  53    -seq_stop 86    -format fasta > XM_002125731.4.fa
esearch -db nuccore -query "NM_131682.2"    | efetch -seq_start  1     -seq_stop 31    -format fasta > NM_131682.2.fa
esearch -db nuccore -query "NM_001035103.2" | efetch -seq_start  7     -seq_stop 40    -format fasta > NM_001035103.2.fa
esearch -db nuccore -query "NM_057753.5"    | efetch -seq_start  78    -seq_stop 111   -format fasta > NM_057753.5.fa
esearch -db nuccore -query "XM_002004955.3" | efetch -seq_start  96    -seq_stop 129   -format fasta > XM_002004955.3.fa
esearch -db nuccore -query "NM_001098.3"    | efetch -seq_start  5     -seq_stop 38    -format fasta > NM_001098.3.fa
esearch -db nuccore -query "XM_844073.5"    | efetch -seq_start  73    -seq_stop 106   -format fasta > XM_844073.5.fa
esearch -db nuccore -query "XM_009859766.3" | efetch -seq_start  145   -seq_stop 178   -format fasta > XM_009859766.3.fa
esearch -db nuccore -query "NM_001174125.2" | efetch -seq_start  1815  -seq_stop 1849  -format fasta > NM_001174125.2.fa
esearch -db nuccore -query "XM_004400823.1" | efetch -seq_start  1843  -seq_stop 1877  -format fasta > XM_004400823.1.fa
esearch -db nuccore -query "XM_013508458.1" | efetch -seq_start  1401  -seq_stop 1435  -format fasta > XM_013508458.1.fa
esearch -db nuccore -query "NM_014585.6"    | efetch -seq_start  95    -seq_stop 128   -format fasta > NM_014585.6.fa
esearch -db nuccore -query "NM_131629.1"    | efetch -seq_start  78    -seq_stop 111   -format fasta > NM_131629.1.fa
esearch -db nuccore -query "NM_001012913.1" | efetch -seq_start  102   -seq_stop 135   -format fasta > NM_001012913.1.fa
esearch -db nuccore -query "NM_003672.4"    | efetch -seq_start  2534  -seq_stop 2567  -format fasta > NM_003672.4.fa
esearch -db nuccore -query "XM_003585862.5" | efetch -seq_start  2189  -seq_stop 2222  -format fasta > XM_003585862.5.fa
esearch -db nuccore -query "NM_001134856.1" | efetch -seq_start  2627  -seq_stop 2660  -format fasta > NM_001134856.1.fa
esearch -db nuccore -query "NM_001430.5"    | efetch -seq_start  70    -seq_stop 104   -format fasta > NM_001430.5.fa
esearch -db nuccore -query "NM_001005647.1" | efetch -seq_start  98    -seq_stop 132   -format fasta > NM_001005647.1.fa
esearch -db nuccore -query "XM_011603052.1" | efetch -seq_start  146   -seq_stop 180   -format fasta > XM_011603052.1.fa
esearch -db nuccore -query "XM_006007491.2" | efetch -seq_start  101   -seq_stop 135   -format fasta > XM_006007491.2.fa
esearch -db nuccore -query "NM_001039806.2" | efetch -seq_start  112   -seq_stop 146   -format fasta > NM_001039806.2.fa
```

Script 2: Unix Script for Retrieval of Sequences in Table S2 Using NCBI Utilities

```
esearch -db nuccore -query "NM_001063.4"   | efetch -seq_start 1     -seq_stop 33     -format fasta > NM_001063.4.fa
esearch -db nuccore -query "S57264.1"      | efetch -seq_start 788   -seq_stop 822    -format fasta > S57264.1.fa
esearch -db nuccore -query "NM_013116.2"   | efetch -seq_start 500   -seq_stop 534    -format fasta > NM_013116.2.fa
esearch -db nuccore -query "M29730.1"      | efetch -seq_start 244   -seq_stop 278    -format fasta > M29730.1.fa
esearch -db nuccore -query "M30785.2"      | efetch -seq_start 1260  -seq_stop 1294   -format fasta > M30785.2.fa
esearch -db nuccore -query "J01611.1"      | efetch -seq_start 2644  -seq_stop 2678   -format fasta > J01611.1.fa
esearch -db nuccore -query "NC_000964.2"   | efetch -seq_start 3913300 -seq_stop 3913334 -format fasta > NC_000964.2.fa
esearch -db nuccore -query "NC_000964.3"   | efetch -seq_start 182348 -seq_stop 182382 -format fasta > NC_000964.3.fa
esearch -db nuccore -query "NM_010403.2"   | efetch -seq_start 1912  -seq_stop 1946   -format fasta > NM_010403.2.fa
esearch -db nuccore -query "NM_005006.6"   | efetch -seq_start 59    -seq_stop 93     -format fasta > NM_005006.6.fa
esearch -db nuccore -query "NM_014117.3"   | efetch -seq_start 58    -seq_stop 92     -format fasta > NM_014117.3.fa
esearch -db nuccore -query "AF059611.1"    | efetch -seq_start 94    -seq_stop 128    -format fasta > AF059611.1.fa
esearch -db nuccore -query "NM_001079802.2" | efetch -seq_start 4538 -seq_stop 4572   -format fasta > NM_001079802.2.fa
esearch -db nuccore -query "NM_000484.4"   | efetch -seq_start 70    -seq_stop 104    -format fasta > NM_000484.4.fa
esearch -db nuccore -query "LR131493.1"    | efetch -seq_start 505294 -seq_stop 505328 -format fasta > LR131493.1.fa
esearch -db nuccore -query "LR131481.1"    | efetch -seq_start 390177 -seq_stop 390211 -format fasta > LR131481.1.fa
esearch -db nuccore -query "LR131482.1"    | efetch -seq_start 187714 -seq_stop 187748 -format fasta > LR131482.1.fa
esearch -db nuccore -query "X90566.1"      | efetch -seq_start 6     -seq_stop 40     -format fasta > X90566.1.fa
esearch -db nuccore -query "XM_027373946.1" | efetch -seq_start 214  -seq_stop 248    -format fasta > XM_027373946.1.fa
esearch -db nuccore -query "DQ207752.1"    | efetch -seq_start 17    -seq_stop 51     -format fasta > DQ207752.1.fa
esearch -db nuccore -query "NM_014826.5"   | efetch -seq_start 7009  -seq_stop 7043   -format fasta > NM_014826.5.fa
esearch -db nuccore -query "NM_079217.3"   | efetch -seq_start 213   -seq_stop 247    -format fasta > NM_079217.3.fa
esearch -db nuccore -query "NG_029489.1"   | efetch -seq_start 108892 -seq_stop 108926 -format fasta > NG_029489.1.fa
esearch -db nuccore -query "AK091913.1"    | efetch -seq_start 1929  -seq_stop 1963   -format fasta > AK091913.1.fa
esearch -db nuccore -query "AK126633.1"    | efetch -seq_start 3183  -seq_stop 3217   -format fasta > AK126633.1.fa
esearch -db nuccore -query "NM_000345.4"   | efetch -seq_start 182   -seq_stop 216    -format fasta > NM_000345.4.fa
esearch -db nuccore -query "NC_000962.3"   | efetch -seq_start 4403226 -seq_stop 4403260 -format fasta > NC_000962.3a.fa
esearch -db nuccore -query "NC_000962.3"   | efetch -seq_start 3023462 -seq_stop 3023496 -format fasta > NC_000962.3b.fa
esearch -db nuccore -query "AY679763.1"    | efetch -seq_start 2722  -seq_stop 2756   -format fasta > AY679763.1.fa
esearch -db nuccore -query "AY371180.1"    | efetch -seq_start 761   -seq_stop 795    -format fasta > AY371180.1.fa
esearch -db nuccore -query "NM_016633.4"   | efetch -seq_start 454   -seq_stop 488    -format fasta > NM_016633.4.fa
esearch -db nuccore -query "XM_017024763.1" | efetch -seq_start 1038 -seq_stop 1072   -format fasta > XM_017024763.1.fa
esearch -db nuccore -query "NM_005329.2"   | efetch -seq_start 3307  -seq_stop 3341   -format fasta > NM_005329.2.fa
esearch -db nuccore -query "NM_001313893.1" | efetch -seq_start 2543 -seq_stop 2577   -format fasta > NM_001313893.1.fa
esearch -db nuccore -query "NM_198560.3"   | efetch -seq_start 4510  -seq_stop 4544   -format fasta > NM_198560.3.fa
esearch -db nuccore -query "NM_012214.3"   | efetch -seq_start 2513  -seq_stop 2547   -format fasta > NM_012214.3.fa
esearch -db nuccore -query "NM_001197026.2" | efetch -seq_start 6823 -seq_stop 6857   -format fasta > NM_001197026.2.fa
esearch -db nuccore -query "NM_000551.4"   | efetch -seq_start 806   -seq_stop 840    -format fasta > NM_000551.4.fa
esearch -db nuccore -query "NM_006870.4"   | efetch -seq_start 238   -seq_stop 272    -format fasta > NM_006870.4.fa
esearch -db nuccore -query "NM_001977.4"   | efetch -seq_start 3082  -seq_stop 3116   -format fasta > NM_001977.4.fa
esearch -db nuccore -query "NM_001080462.3" | efetch -seq_start 600  -seq_stop 634    -format fasta > NM_001080462.3.fa
esearch -db nuccore -query "NM_001082968.2" | efetch -seq_start 359  -seq_stop 393    -format fasta > NM_001082968.2.fa
esearch -db nuccore -query "NM_001025573.2" | efetch -seq_start 954  -seq_stop 988    -format fasta > NM_001025573.2.fa
esearch -db nuccore -query "NM_178629.6"   | efetch -seq_start 2143  -seq_stop 2177   -format fasta > NM_178629.6.fa
esearch -db nuccore -query "NM_173737.2"   | efetch -seq_start 133   -seq_stop 167    -format fasta > NM_173737.2.fa
esearch -db nuccore -query "NM_001081436.2" | efetch -seq_start 1060 -seq_stop 1094   -format fasta > NM_001081436.2.fa
esearch -db nuccore -query "AK134743.1"    | efetch -seq_start 818   -seq_stop 852    -format fasta > AK134743.1.fa
esearch -db nuccore -query "NM_173505.4"   | efetch -seq_start 1945  -seq_stop 1979   -format fasta > NM_173505.4.fa
esearch -db nuccore -query "NM_183170.2"   | efetch -seq_start 162   -seq_stop 196    -format fasta > NM_183170.2.fa
esearch -db nuccore -query "NM_023158.7"   | efetch -seq_start 1342  -seq_stop 1376   -format fasta > NM_023158.7.fa
esearch -db nuccore -query "NM_011807.3"   | efetch -seq_start 2320  -seq_stop 2354   -format fasta > NM_011807.3.fa
esearch -db nuccore -query "NM_001364.3"   | efetch -seq_start 4669  -seq_stop 4703   -format fasta > NM_001364.3.fa
esearch -db nuccore -query "NM_010118.3"   | efetch -seq_start 1238  -seq_stop 1272   -format fasta > NM_010118.3.fa
esearch -db nuccore -query "NR_028406.2"   | efetch -seq_start 469   -seq_stop 503    -format fasta > NR_028406.2.fa
esearch -db nuccore -query "NM_019994.5"   | efetch -seq_start 5172  -seq_stop 5206   -format fasta > NM_019994.5.fa
esearch -db nuccore -query "NM_001003719.2" | efetch -seq_start 710  -seq_stop 744    -format fasta > NM_001003719.2.fa
esearch -db nuccore -query "NM_008184.3"   | efetch -seq_start 1084  -seq_stop 1118   -format fasta > NM_008184.3.fa
esearch -db nuccore -query "NM_133994.3"   | efetch -seq_start 955   -seq_stop 989    -format fasta > NM_133994.3.fa
esearch -db nuccore -query "NM_013755.4"   | efetch -seq_start 473   -seq_stop 507    -format fasta > NM_013755.4.fa
esearch -db nuccore -query "NM_004130.4"   | efetch -seq_start 462   -seq_stop 496    -format fasta > NM_004130.4a.fa
esearch -db nuccore -query "NM_004130.4"   | efetch -seq_start 1275  -seq_stop 1309   -format fasta > NM_004130.4b.fa
esearch -db nuccore -query "NM_201531.4"   | efetch -seq_start 3177  -seq_stop 3211   -format fasta > NM_201531.4a.fa
esearch -db nuccore -query "NM_201531.4"   | efetch -seq_start 4720  -seq_stop 4754   -format fasta > NM_201531.4b.fa
esearch -db nuccore -query "NM_013587.3"   | efetch -seq_start 2272  -seq_stop 2306   -format fasta > NM_013587.3.fa
esearch -db nuccore -query "NM_173199.3"   | efetch -seq_start 387   -seq_stop 421    -format fasta > NM_173199.3.fa
esearch -db nuccore -query "NM_016467.5"   | efetch -seq_start 894   -seq_stop 928    -format fasta > NM_016467.5.fa
esearch -db nuccore -query "NM_001101479.1" | efetch -seq_start 1151 -seq_stop 1185   -format fasta > NM_001101479.1.fa
esearch -db nuccore -query "NM_144828.2"   | efetch -seq_start 573   -seq_stop 607    -format fasta > NM_144828.2.fa
esearch -db nuccore -query "NM_024854.5"   | efetch -seq_start 938   -seq_stop 972    -format fasta > NM_024854.5.fa
esearch -db nuccore -query "NM_021202.3"   | efetch -seq_start 2043  -seq_stop 2077   -format fasta > NM_021202.3.fa
esearch -db nuccore -query "NM_019863.2"   | efetch -seq_start 8     -seq_stop 42     -format fasta > NM_019863.2.fa
esearch -db nuccore -query "NM_020139.4"   | efetch -seq_start 2276  -seq_stop 2310   -format fasta > NM_020139.4.fa
esearch -db nuccore -query "NM_002628.4"   | efetch -seq_start 750   -seq_stop 784    -format fasta > NM_002628.4.fa
esearch -db nuccore -query "NM_007824.3"   | efetch -seq_start 3086  -seq_stop 3110   -format fasta > NM_007824.3.fa
esearch -db nuccore -query "NM_145005.6"   | efetch -seq_start 104   -seq_stop 138    -format fasta > NM_145005.6.fa
esearch -db nuccore -query "NW_001915125.1" | efetch -seq_start 2650 -seq_stop 2684   -format fasta > NW_001915125.1a.fa
esearch -db nuccore -query "NW_001915125.1" | efetch -seq_start 3355 -seq_stop 3382   -format fasta > NW_001915125.1b.fa
esearch -db nuccore -query "NM_012334.3"   | efetch -seq_start 4649  -seq_stop 4683   -format fasta > NM_012334.3.fa
esearch -db nuccore -query "XM_011511385.3" | efetch -seq_start 614  -seq_stop 648    -format fasta > XM_011511385.3.fa
esearch -db nuccore -query "MN339453.1"    | efetch -seq_start 2961  -seq_stop 2995   -format fasta > MN339453.1.fa
esearch -db nuccore -query "MN339467.1"    | efetch -seq_start 1869  -seq_stop 1903   -format fasta > MN339467.1.fa
```
